# Exosomal long noncoding RNA HOXD-AS1 promotes prostate cancer metastasis via miR-361-5p/FOXM1 axis

**DOI:** 10.1038/s41419-021-04421-0

**Published:** 2021-12-04

**Authors:** Yongming Jiang, Hui Zhao, Yuxiao Chen, Kangjian Li, Tianjie Li, Jianheng Chen, Baiyu Zhang, Caifen Guo, Liangliang Qing, Jihong Shen, Xiaodong Liu, Peng Gu

**Affiliations:** 1 Department of Urology, The 1st Affiliated Hospital of Kunming Medical University, Kunming, 650032 China; 2 Department of Urology, The 2nd Affiliated Hospital of Kunming Medical University, Kunming, 650101 China; 3 Yunnan Province Clinical Research Center for Chronic Kidney Disease, Kunming, 650032 China; 4 Department of Urology, The Second People’s Hospital of Qujing City, Qujing City, Yunnan Province 655000 China

**Keywords:** Bone metastases, Prostate cancer

## Abstract

Development of distant metastasis is the main cause of deaths in prostate cancer (PCa) patients. Understanding the mechanism of PCa metastasis is of utmost importance to improve its prognosis. The role of exosomal long noncoding RNA (lncRNA) has been reported not yet fully understood in the metastasis of PCa. Here, we discovered an exosomal lncRNA HOXD-AS1 is upregulated in castration resistant prostate cancer (CRPC) cell line derived exosomes and serum exosomes from metastatic PCa patients, which correlated with its tissue expression. Further investigation confirmed exosomal HOXD-AS1 promotes prostate cancer cell metastasis in vitro and in vivo by inducing metastasis associated phenotype. Mechanistically exosomal HOXD-AS1 was internalized directly by PCa cells, acting as competing endogenous RNA (ceRNA) to modulate the miR-361-5p/FOXM1 axis, therefore promoting PCa metastasis. In addition, we found that serum exosomal HOXD-AS1 was upregulated in metastatic PCa patients, especially those with high volume disease. And it is correlated closely with Gleason Score, distant and nodal metastasis, Prostatic specific antigen (PSA) recurrence free survival, and progression free survival (PFS). This sheds a new insight into the regulation of PCa distant metastasis by exosomal HOXD-AS1 mediated miR-361-5p/FOXM1 axis, and provided a promising liquid biopsy biomarker to guide the detection and treatment of metastatic PCa.

## Introduction

Prostate cancer (PCa) is the second commonly diagnosed malignancy and one of the leading cause of male cancer-related death worldwide [1]. Metastatic PCa is treated with either antiandrogen or chemotherapy regimens on the basis of androgen deprivation. Despite almost all patients respond to the initial treatment, disease progression is often inevitable after 18–24 months [2]. However, the mechanism of PCa metastasis is not fully understood. Accumulating evidence support the theories that pre-existing castration resistant PCa cells, as well as adaptive genetic or epigenetic alteration, concomitantly contribute to the metastasis of PCa [3–5]. Recent studies also revealed that tumor microenvironment (TME), which promotes the conversion of PCa cell phenotypes through various ways, plays important roles in the metastasis of PCa [6, 7]. However, the mechanism underlying how PCa cells acquire metastatic features during evolving remains elusive. Identifying novel molecular mechanisms of how TME remold PCa phenotypes during metastasis holds great promise to improve the diagnosis and treatment of metastatic PCa.

Exosomes are membranous microvesicles ranging 40–150 nm in dimension, which are found in various human fluids including, but not limited to, blood, urine, and bile [8]. Recently, tumor cell-derived exosomes are recognized as messengers that modulate local and systemic TME by transferring bioactive molecules such as proteins, RNAs, and DNAs [9]. Notably, long non-coding RNAs(lncRNAs) are identified as key molecular cargos of tumor cell-derived exosomes [10]. These functional lncRNAs transported by exosomes to a recipient cell can regulate tumor metastasis and progression by modulating downstream gene expression [11, 12]. Although recent studies revealed that TME derived exosomes exerts important regulatory role in PCa progression [13, 14], the biological function and mechanism of cancer cell-secreted exosomes in the distant metastasis of PCa remains unclear, warranting further exploration.

Previously we constructed two castration resistant prostate cancer (CRPC) cell models, LNCaP-AI and LNCaP-Bic, by exposing LNCaP cells to continuous androgen deprived medium or antiandrogen drug. These two cell lines displayed similar biological and molecular characteristics with clinical CRPC [15]. We demonstrated that the lncRNA HOXD-AS1 is an important regulator in the progression of PCa by using these two models [15]. Herein, we reported that HOXD-AS1 was found overexpressed in these cell derived exosomes and serum exosomes from PCa patients, which correlated with distant metastasis and survival. Functionally, exosomal HOXD-AS1 promoted migration in vitro, and distant metastasis of prostate cancer cell in vivo. Mechanistically, exosomal HOXD-AS1 was transferred directly to PCa cells, in which HOXD-AS1 served as competing endogenous RNA (ceRNA) by sponging miR-361-5p, which upregulated the expression of Forkhead box M1 (FOXM1), therefore facilitating metastasis. Our findings highlight the mechanism of exosomal HOXD-AS1 mediated transmitting of metastatic features in the PCa TME, and identified exosomal HOXD-AS1 as a potential marker of liquid biopsy for metastasis in PCa.

## Results

### HOXD-AS1 is overexpressed in LNCaP-Bic and LNCaP-AI cell-derived and PCa patients’ serum exosomes

**Fig. 1:**
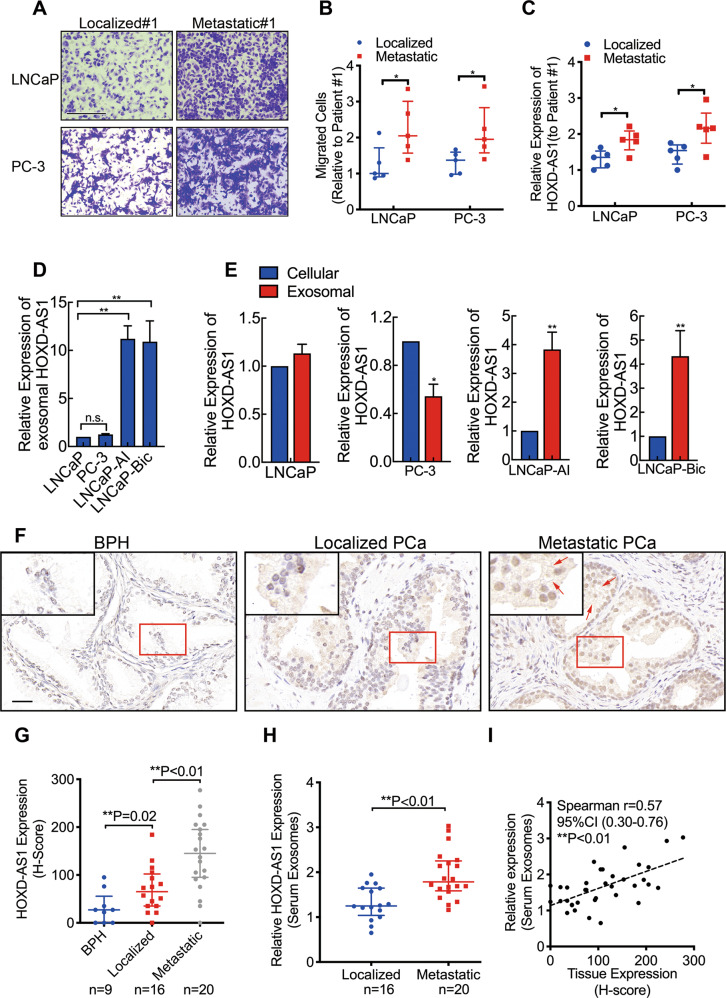
HOXD-AS1 is overexpressed in LNCaP-Bic and LNCaP-AI cell-derived and PCa patients’ serum exosomes. **A**, **B** LNCaP and PC-3 cells were cultured with medium supplied with serum exosomes enriched from localized and metastatic PCa patients (each *n* = 5) for 48 h, and transwell migration assay was used to measure the migration. Representative image of localized and metastatic patient #1 was displayed. The results were displayed as relative ratio to patient #1, presented as the medians ± interquartile of values obtained from experiments. Scale bar: 200 μm. **C** LNCaP and PC-3 cells were cultured with medium supplied with serum exosomes enriched from localized and metastatic PCa patients (each *n* = 5) for 48 h, then the HOXD-AS1 expression was detected using real-time qPCR. The results were normalized to GAPDH and presented as relative value to that of patient #1, displayed as medians ± interquartile of values. **D** LNCaP, PC-3, LNCaP-AI, and LNCaP-Bic cells were cultured for 48 h and exosomes from cultural medium was collected, the exosomal HOXD-AS1 expression was detected by qPCR. The results of real time qPCR were normalized to GAPDH and presented as the means ± SD of values obtained in three independent experiments. **E** Cellular and exosomal HOXD-AS1 expression was compared in different PCa cells, as detected by qPCR. The results of real-time qPCR were normalized to respective GAPDH and presented as the means ± SD of values obtained in three independent experiments. **F** Representative images of RNA in situ hybridization (RNA-ISH) of HOXD-AS1 expression (brown) in paraffin-embedded BPH (*n* = 9) and PCa (*n* = 36, 16 localized and 20 metastatic) tissue. Scale bars: 50 μm. Red arrows indicate extracellular signals. **F** RNA-ISH of HOXD-AS1 expression was quantified by the expression score (0–300). The results are presented as medians ± interquartile. **G** Serum exosome was enriched from PCa patients (*n* = 36, 16 localized and 20 metastatic) and exosomal HOXD-AS1 was detected by PCR. The results of real time qPCR were normalized to GAPDH and displayed as median ± interquartile. **H** Correlation of HOXD-AS1 expression between PCa tissue and serum exosomes was analyzed by Spearman correlation. Exosomes were normalized by identical protein quantity. *p < 0.05, **p < 0.01. See also Figs. S1–2.

To investigate the effect of serum exosomes from PCa patients on PCa cells, we treated LNCaP and PC-3 cells with exosomes from localized and metastatic patients (each *n* = 5). Surprisingly, we found that the serum exosomes from metastatic patients significantly enhanced the motility of PCa cells, as evaluated by transwell and wound healing assays (Fig. 1A, B, Fig. S1A, B and S2A–C). Notably, an increased expression of HOXD-AS1 was observed in the cells treated with metastatic patients’ serum exosomes (Fig. 1C). Then we applied LNCaP-Bic and LNCaP-AI cells for further study. We found that HOXD-AS1 was significantly overexpressed in the LNCaP-Bic and LNCaP-AI derived exosomes, as compared with those from LNCaP and PC-3 (Fig. 1D). Additionally, we also noticed that HOXD-AS1 was highly enriched in LNCaP-Bic and LNCaP-AI enriched exosomes than that of cellular expression, but not LNCaP and PC-3 (Fig. 1E). Next, RNA in-situ hybridization found that HOXD-AS1 was overexpressed in metastatic PCa specimens (Fig. 1F, G). Notably, extra-cellular expression of HOXD-AS1 was also observed, further indicating the existence of exosomal HOXD-AS1 in clinical specimens (Fig. 1F). Moreover, HOXD-AS1 was found significantly upregulated in metastatic PCa patients (Fig. 1H). Interestingly, we also noticed that HOXD-AS1 expression in serum exosomes was closely correlated with its tissue expression in PCa patients (Fig. 1I, *r* = 0.57, *P* < 0.01). Collectively, these findings indicate that exosomal HOXD-AS1 may participated in the metastasis of PCa.

### LNCaP-Bic and LNCaP-AI cell-derived exosomes promote PCa cell migration in vitro by inducing metastasis associated phenotype

**Fig. 2:**
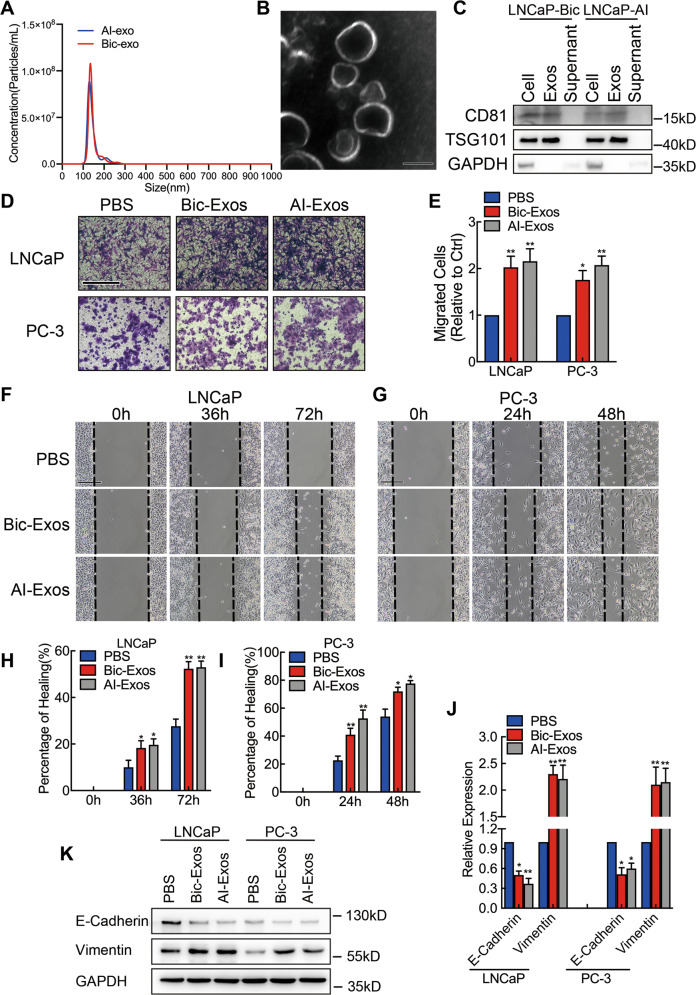
LNCaP-Bic and LNCaP-AI cell-derived exosomes promote PCa cell migration in vitro by inducing metastasis associated phenotype. **A** Purified exosomes from LNCaP-AI and LNCaP-Bic cells were analyzed by NanoSight. **B** Representative image of CRPC-Exos under TEM, scale bar: 100 nm. **C** Western Blot analysis of exosome markers CD81 and TSG101 in LNCaP-Bic and LNCaP-AI cell lysate, exosomes, and supernant. **D**, **E** LNCaP and PC-3 cells were treated with purified LNCaP-Bic and LNCaP-AI exosomes for 48 h and the migration ability was measured by transwell assays. The results were displayed as relative ratio to control, presented as the means ± SD of values obtained in three independent experiments. Scale bar: 200 μm. **F**–**I** LNCaP and PC-3 cells were treated with purified LNCaP-Bic and LNCaP-AI exosomes for 48 h and the cellular motility was evaluated by wound healing assay. Wound healing was measured by the percentage of healing compared with baseline and presented as means ± SD of values obtained in three independent experiments. Scale bar: 100 μm. **J**, **K** E-Cadherin and Vimentin was detected as metastatic phenotype related marker by qPCR and Wstern Blot. The results of real time qPCR were normalized to GAPDH and presented as the means ± SD of values obtained in three independent experiments. Exosomes were normalized by identical protein quantity. *p < 0.05, **p < 0.01. See also Fig. S3.

First of all, we found that the motility of LNCaP and PC-3 cell was enhanced significantly when cultured with LNCaP-Bic and LNCaP-AI-conditioned medium (Fig. S3A, B). Then exosomes derived by LNCaP-Bic and LNCaP-AI cells were isolated from cultural medium. We found that exosomes with an 80–150 nm in size and a typical cup-shaped morphology were detected by NanoSight analysis (NTA) (Fig. 2A) and transmission electron microscopy (TEM) (Fig. 2B). Then exosomal protein markers CD81 and tumor susceptibility 101 (TSG101) were detected by Western Blot, these markers were detectable from both cell lysate and exosomes, but not the supernant. This result further confirmed that the particles enriched from the culture medium were exosomes (Fig. 2C). Consistent with our findings, these two exosomes also enhanced the motility of PCa cells, as measured by transwell (Fig. 2D, E) and wound healing assays (Fig. 2F–I). Additionally, we investigated if this phenomenon was associated with epithelial to mesenchymal transition (EMT). As expected, we confirmed that the epithelial marker E-Cadherin was downregulated, while the mesenchymal marker Vimentin was upregulated after PCa cells were treated with LNCaP-Bic and LNCaP-AI exosomes (Fig. 2J, K). Above all, our data demonstrate that LNCaP-Bic and LNCaP-AI secreted exosomes promoted migration of PCa in vitro by inducing metastatic phenotype.

### CRPC derived exosome enhances PCa cell motility by delivering HOXD-AS1

**Fig. 3:**
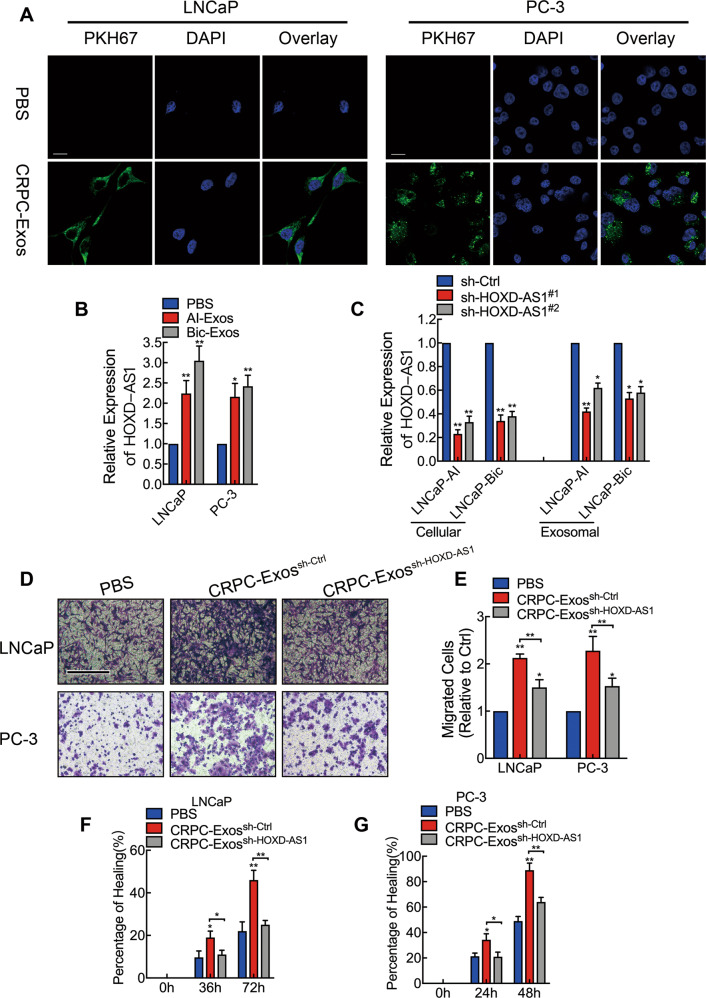
CRPC derived exosome enhances PCa cell motility by delivering HOXD-AS1. **A** CRPC cell secreted exosomes were labeled with PKH67(green) and incubated with PCa cells for 24 h, images were captured by a confocal microscope, equal amount of PBS was used as negative control. Scale bar: 10 μm. **B** LNCaP and PC-3 cells were incubated with purified LNCaP-Bic and LNCaP-AI exosomes for 48 h and cellular expression of HOXD-AS1 was detected by qPCR. The results of real time qPCR were normalized to GAPDH and presented as the means ± SD of values obtained in three independent experiments. **C** Stable HOXD-AS1 knockdown LNCaP-AI and LNCaP-Bic cells were constructed by lentiviral transduction, cellular and correspondent exosomal HOXD-AS1 was detected by qPCR. The results of real-time qPCR were normalized to GAPDH and presented as the means ± SD of values obtained in three independent experiments. **D**, **E** LNCaP and PC-3 cells were treated with either purified HOXD-AS1 knockdown exosomes or control exosomes for 48 h, and transwell assay was conducted to measure the cellular migration, PBS were used as negative control. The results were displayed as relative ratio to control, presented as the means ± SD of values obtained in three independent experiments. Scale bar: 200 μm. **F**, **G** LNCaP and PC-3 cells were treated with either purified HOXD-AS1 knockdown exosomes or control exosomes for 48 h, then cellular motility was evaluated by wound healing assay, PBS were used as negative control. Wound healing was measured by the percentage of healing compared with baseline and presented as means ± SD of values obtained in three independent experiments. LNCaP-AI derived exosomes were used to represent CRPC cell-derived exosomes for study and mentioned as CRPC-Exos, exosomes were normalized by identical protein quantity. Scale bar: 100 μm. *p < 0.05, **p < 0.01. See also Fig. S4.

We then tried to investigate whether this biological function was achieved through transferring of HOXD-AS1 by exosomes. As these two types of exosomes displayed similar characteristics and biological effect, we used LNCaP-AI secreted exosomes for further study and mentioned as CRPC-Exos afterward. First of all, we labeled purified exosomes with PKH67 green fluorescent dye and incubated them with PCa cells for 24 h. Confocal images showed the green fluorescent punctate signal in the cytoplasm of recipient PCa cells, indicating internalization of the PKH67-labeled exosomes. By contrast, no PKH67 fluorescent signal was observed in the control group, suggesting that the PCa cells specifically internalized the CRPC-Exos (Fig. 3A). Then we found that HOXD-AS1 expression was significantly elevated in PCa cells after incubated with the CRPC-Exos (Fig. 3B). Moreover, HOXD-AS1 knockdown in LNCaP-AI and LNCaP-Bic cells led to a significant reduced (Fig. 3C), while HOXD-AS1 overexpression resulted in an increased HOXD-AS1 expression in respective exosomes (Fig. S4A). Furthermore, exosomes from HOXD-AS1 knockdown LNCaP-Bic and LNCaP-AI cells failed to enhance HOXD-AS1 expression when treated to PCa cells (Fig. S4B, C). Moreover, we found that incubating CRPC-Exos with HOXD-AS1 knockdown PCa cells reversed the effect of HOXD-AS1 downregulation (Fig. S4D, E). As a consequence, HOXD-AS1 knockdown in CRPC-Exos downregulated TSG101 expression, diminished its ability of enhancing PCa cell motility, as measured by transwell (Fig. 3D, E) and wound healing assays (Fig. 3F, G, Fig. S4F, G). Taken together, our data demonstrated that CRPC-Exos promoted the motility of PCa cells by transmitting HOXD-AS1.

### CRPC cell secreted exosomal HOXD-AS1 promotes distant metastasis of PCa in vivo

**Fig. 4:**
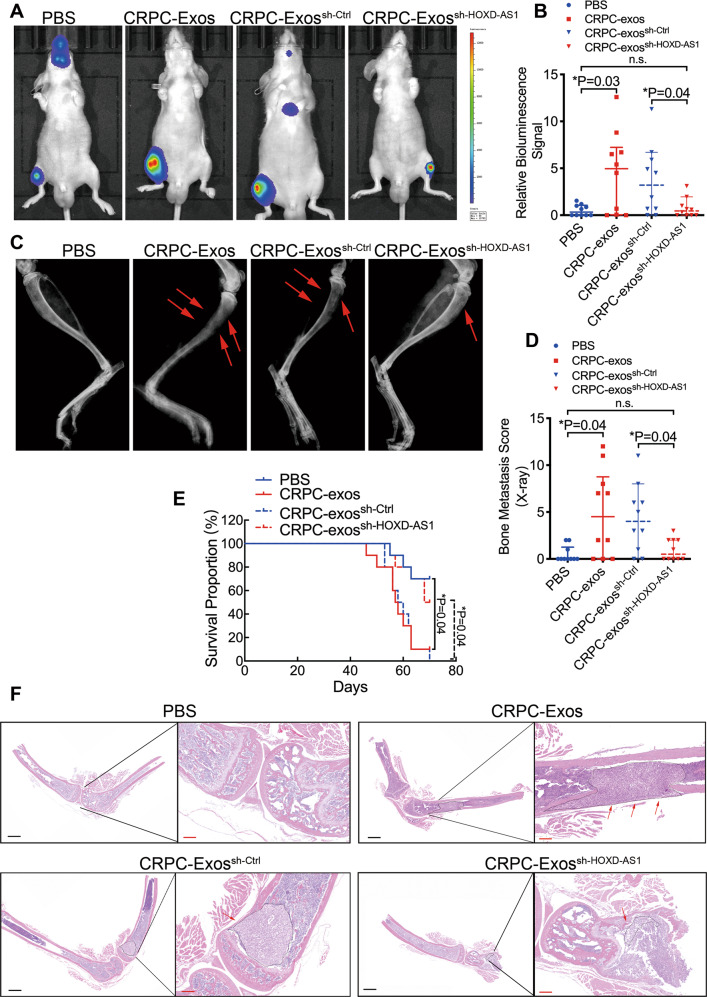
Exosomal HOXD-AS1 promotes distant metastasis of PCa in vivo. **A** PC-3 cells were pre-treated with either PBS or CRPC-Exos, HOXD-AS1 knockdown CRPC-Exos or respective control for 48 h and injected intra-cardiac to mimic the process of bone metastasis. Representative bioluminescence images of bone metastasis of a mouse at 8 weeks were displayed. **B** Quantification of the bioluminescence imaging signal in the PBS and CRPC-Exos groups, or HOXD-AS1 knockdown CRPC-Exos and its control at 8 weeks (each *n* = 10). The results are presented as medians ± interquartile. **C** Representative radiographic images of bone metastasis in the indicated mice (arrows indicate osteolytic lesions). **D** The sum of bone metastasis scores for each mouse in tumor-bearing mice in each group (each *n* = 10). The results are presented as medians ± interquartile. **E** Kaplan–Meier analysis of bone metastasis-free survival in each group. **F** Representative images of H&E-stained sections of tibias from the indicated mouse. Arrows indicate the osteolytic lesions. Black dot-circled areas indicate the metastatic tumor in the bone. Black scale bars: 2000 μm, red scale bars: 500 μm. Exosomes were normalized by identical protein quantity. *p < 0.05, **p < 0.01. See also Fig. S5.

To further explore the function of exosomal HOXD-AS1 in the metastasis of PCa, we applied a mouse model of bone metastasis. First of all, luciferase-expressing PC-3 cells were pre-treated with either PBS or CRPC-Exos, HOXD-AS1 knockdown CRPC-Exos or its control for 48 h and then inoculated into the left cardiac ventricle of male nude mice. After the inoculation, respective exosomes were injected intra-cardiac weekly to ensure a constant effect of exosomes on PCa cells. Surprisingly, CPRC-Exos strongly promoted the formation of bone metastasis, as detected by bioluminescence imaging (Fig. 4A, B). By contrast, the pro-metastatic feature of CRPC-Exos was significantly diminished by HOXD-AS1 knockdown (Fig. 4A, B). The bone metastasis was further confirmed by X-ray imaging, indicating a significantly worse destruction of cortical bone and higher bone score in the CRPC-Exos group, but not the group treated with HOXD-AS1 knockdown exosomes (Fig. 4C, D). Meanwhile, a significantly shortened metastatic-free survival was observed in the CRPC-Exos group, as compared with PBS (Fig. 4E). And downregulation of HOXD-AS1 in CRPC-Exos significantly prolonged the survival of indicated mouse, as compared with control (Fig. 4E). Furthermore, H&E staining on the bone tissue sections indicated an increased bone metastasis burden and more extensive osteolytic lesions in the CRPC-Exos treated group (Fig. 4F). Consistently, significant less metastatic tumor and osteolytic lesions were observed in the group using HOXD-AS1 knockdown exosomes. Finally, immunohistochemistry (IHC) with anti-firefly luciferase antibody further confirmed the metastatic sites (Fig. S5). Collectively, our results supported that CRPC cell derived exosomal HOXD-AS1 promoted the distant metastasis of PCa in vivo.

### Exosomal HOXD-AS1 promotes PCa metastasis via miR-361-5p/FOXM1 axis

**Fig. 5:**
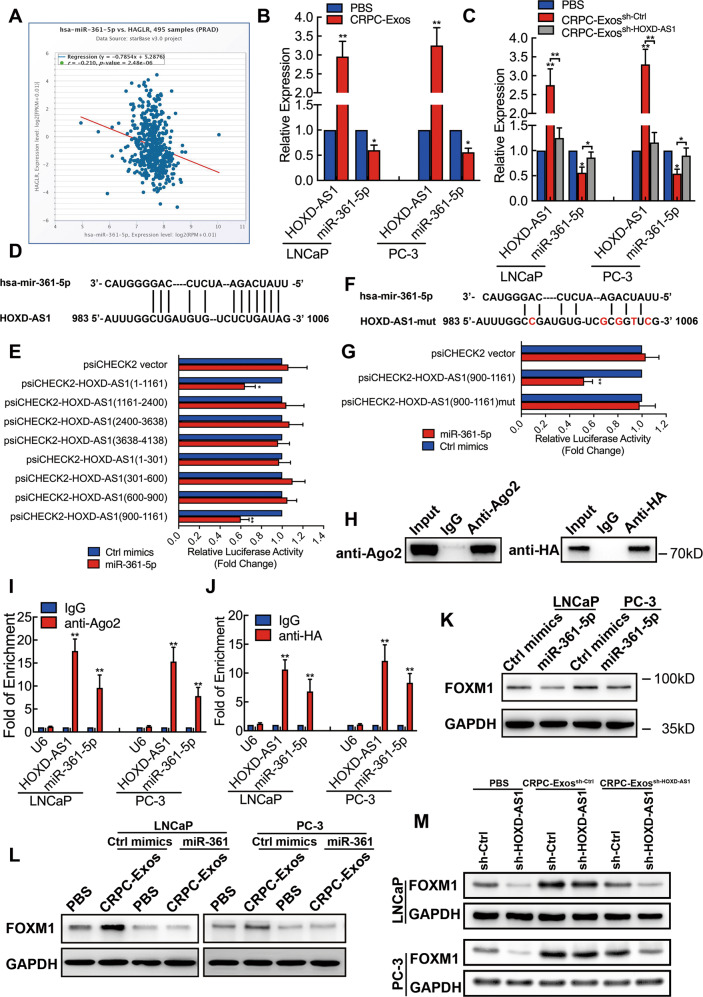
Exosomal HOXD-AS1 promotes PCa metastasis via miR-361-5p/FOXM1 axis. **A** The correlation of HOXD-AS1 and miR-361-5p expression in TCGA PRAD cohort was analyzed by starBase. **B** LNCaP and PC-3 cells were incubated with purified CRPC-Exos for 48 h, the expression of HOXD-AS1 and miR-361-5p was detected by qPCR. The results are presented as the means ± SD of values obtained in three independent experiments. **C** LNCaP and PC-3 cells were treated with HOXD-AS1 knockdown CRPC-Exos or control exosomes for 48 h, the expression of HOXD-AS1 and miR-361-5p was detected by qPCR. The results are presented as the means ± SD of values obtained in three independent experiments. **D** An illustration of the binding site between HOXD-AS1 with miR-361-5p, predicted by miRanda and starBase. **E** Different truncated HOXD-AS1 fragment was cloned into psiCHECK2 vector, and luciferase reporter assay was conducted. Empty psiCHECK2 vector was used as negative control. The results are presented as the means ± SD of values obtained in three independent experiments. **F** A schematic illustration of site-directed mutagenesis on the HOXD-AS1 and miR-361-5p binding site. **G** Luciferase reporter assay was conducted using wild type or mutated HOXD-AS1 and miR-361-5p binding site. Empty psiCHECK2 vector was used as negative control. The results are presented as the means ± SD of values obtained in three independent experiments. (**H**–**J**). RNA immunoprecipitation using either anti-Ago2 or anti-HA was conducted, the enrichment of Ago2 or HA was detected by Western Blot. The expression of HOXD-AS1 and miR-361-5p from the product was detected by qPCR. RNA enrichment was determined relative to the non-immuned IgG control. U6 was used as a non-specific control. The results are presented as the means ± SD of values obtained in three independent experiments. **K** LNCaP and PC-3 cells were transfected with miR-361-5p mimics or control mimics for 48 h, and FOXM1 was detected by Western Blot, GAPDH was used as an internal control. **L** LNCaP and PC-3 cells were pre-treated with CRPC-Exos for 48 h, then transfected with either miR-361-5p mimics or control mimics. FOXM1 expression was evaluated by Western Blot, GAPDH was used as an internal control. **M** HOXD-AS1 depleted LNCaP and PC-3 cells were treated with either CRPC-Exos or HOXD-AS1 knockdown CRPC-Exos for 48 h, FOXM1 was detected by Western Blot, GAPDH was used as an internal control. Exosomes were normalized by identical protein quantity. For real-time qPCR results, HOXD-AS1 expression was normalized to GAPDH and miR-361-5p expression was normalized to U6. *p < 0.05, **p < 0.01. See also Figs. S6–7.

Considering our findings that CRPC-Exos was internalized in the cytoplasm, we sought to investigate whether exosomal HOXD-AS1 function as RNA sponge in PCa cells. Interestingly, as one of the most enriched mircoRNA in PCa, the expression of miR-361-5p was negatively correlated with HOXD-AS1 (*R* = −0.21, *P* < 0.01), as analyzed by Starbase database (Fig. 5A) [16]. The correlation was strong when compared with other published studies (Fig. S6A–E, Supplementary refs. 1–5). Notably, miR-361-5p is an important tumor suppressor [17–19] and inhibit metastasis through inhibiting EMT in prostate cancer [20]. We then proposed that exosomal HOXD-AS1 may interacted with miR-361-5p after it was delivered by exosomes. To test our hypothesis, we treated PCa cells with CRPC-Exos and found that the expression of miR-361-5p was significantly downregulated, accompanied by the upregulation of HOXD-AS1 (Fig. 5B). On the other hand, the enhanced miR-361-5p expression by HOXD-AS1 knockdown in PCa cells could be impaired through treating with CRPC-Exos (Fig. S6F, G). Similar with the effect of CRPC-Exos, overexpression of HOXD-AS1 inhibited the expression (Fig. S6H), while downregulation of HOXD-AS1 upregulated miR-361-5p in PCa cells (Fig. S6I). On the other hand, exosomes from HOXD-AS1 knockdown CRPC cells were unable to inhibit the expression of miR-361-5p (Fig. 5C). Furthermore, by searching miRanda and CLIP-seq data from Starbase [16], we identified a potential miR-361-5p binding site at 983–1006nt of HOXD-AS1 (Fig. 5D). PsiCHECK2 vector containing segment of HOXD-AS1 was generated, then luciferase assay was conducted to identify the region of HOXD-AS1 binding with mir-361-5p. Consistent with bioinformatic prediction, we found that the luciferase activity was significantly inhibited with the segment containing HOXD-AS1 983~1006nt, but not other regions (Fig. 5E). Next, vector containing site-directed mutagenesis of miR-361-5p binding site was constructed (Fig. 5F). MiR-361-5p significantly inhibited the luciferase activity of the vector containing wild-type HOXD-AS1 fragment, but not the mutant vector (Fig. 5G). Additionally, we performed an RNA immunoprecipitation (RIP) and found a significant enrichment of both HOXD-AS1 and miR-361-5p by argonaute RISC catalytic component 2 (Ago2) antibody compared with IgG (Fig. 5H, I). Besides, we also observed that HOXD-AS1 and miR-361-5p was able to be enriched by exogeneous Ago2, as detected by RIP using Ago2 with HA tag (Fig. 5H, J), which further supported their specific interaction. Furthermore, we detected the expression of *FOXM1*, a key modulator in prostate cancer progression and metastasis, as well as the most reported miR-361-5p target [19, 21]. As a result, FOXM1 expression was significantly inhibited by miR-361-5p transfection in PCa cells, as detected by Western Blot (Fig. 5K). CRPC-Exos significantly increased the expression of FOXM1 in PCa cells, while overexpression of miR-361-5p reversed its effect (Fig. 5L). Interestingly, while knockdown of HOXD-AS1 in PCa cells resulted in a significantly reduced expression of FOXM1, treating the cells with CRPC-Exos obviously upregulated FOXM1 expression, and reversed the downregulation of FOXM1 caused by HOXD-AS1 depletion (Fig. 5M). By contrast, HOXD-AS1 downregulated CRPC-Exos were unable to compensate the effect of HOXD-AS1 depletion in PCa cells (Fig. 5M). Last but not least, we observed that while CRPC-Exos strongly promoted the migration ability of PCa cells, FOXM1 silencing impaired the effect (Fig. S7A–F). These results suggest that the enhanced migration by CRPC-Exos was achieved through FOXM1. Taken together, our data clearly demonstrated that exosomal HOXD-AS1 function as an ceRNA sponging miR-361-5p, which in turn upregulated the expression of FOXM1 in PCa cells, therefore promoting distant metastasis.

### Serum exosomal HOXD-AS1 expression associates with clinical characteristics and prognosis in PCa

**Fig. 6:**
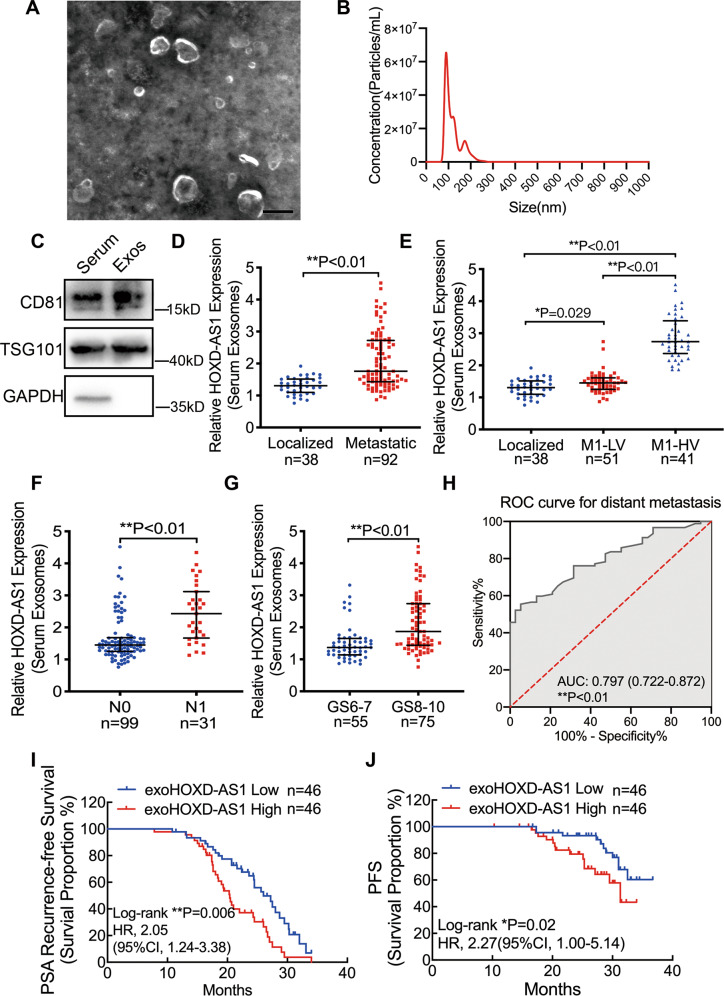
Serum exosomal HOXD-AS1 expression associates with clinical characteristics and prognosis in PCa. **A** Representative image of serum exosomes from PCa patients under TEM, scale bar: 100 nm. **B** Purified serum exosomes from PCa patients were analyzed by NanoSight. **C** Western Blot analysis of exosome markers CD81 and TSG101 in PCa patients’ serum and serum exosomes. **D** The serum exosomal HOXD-AS1 expression from PCa patients was detected by qPCR (total *n* = 130, localized *n* = 38, metastatic *n* = 92). HOXD-AS1 expression was normalized to GAPDH, and displayed as relative expression. The results are presented as medians ± interquartile. **E** The relative expression of serum exosomal HOXD-AS1 in localized, low-volume metastatic and high-volume metastatic (indicated as M1-LV and M1-HV, respectively) PCa patients (*n* = 38, 51, 41, respectively). The results are presented as medians ± interquartile. **F** The relative expression of serum exosomal HOXD-AS1 between non-lymph node metastasis (N0) and lymph node positive (N1) PCa patients (*n* = 99 and 31). The results are presented as medians ± interquartile. **G** The relative expression of serum exosomal HOXD-AS1 between Gleason Score 6–7 and Gleason Score 8–10 PCa patients (*n* = 55 and 75). The results are presented as medians ± interquartile. **H** ROC curve analysis for evaluating the diagnostic potential of serum exosomal HOXD-AS1 for distant metastasis. **I**, **J** The PSA recurrence-free survival and progression-free survival rates of the metastatic PCa patients were compared by Kaplan–Meier analysis in the serum exosomal HOXD-AS1-low and high groups. Median expression was used as cut off value in the survival analysis (*n* = 92). Exosomes were normalized by identical protein quantity. *p < 0.05, **p < 0.01. See also Fig. S8–9.

To explore the clinical significance of serum exosomal HOXD-AS1, we first isolated exosomes from the serum of treatment-naïve PCa patients and characterized its features, which was similar with our findings from cellular exosomes on morphology, dimension (Fig. 6A, B) and protein markers (Fig. 6C). Secondly, we isolated the serum exosomes of a PCa cohort with 130 patients before their initial treatment and detected the expression the HOXD-AS1 by qPCR. Serum exosomal HOXD-AS1 was significantly elevated in metastatic PCa patients, as compared with that of localized ones (Fig. 6D). Interestingly, the expression of serum exosomal HOXD-AS1 was much more obviously increased in M1 patients with high metastatic volume compared with either low volume or localized disease (Fig. 6E). Besides, serum exosomal HOXD-AS1 expression was also significantly upregulated in PCa patients with positive nodal metastasis and higher Gleason Score (Fig. 6F, G), but not tumor stage (Fig. S8). Meanwhile, serum exosomal HOXD-AS1 level was significantly correlated with the Gleason Score, lymph node, and metastatic status of PCa patients (Table 1). Receiver operating characteristic (ROC) analysis showed that both serum exosomal HOXD-AS1 and PSA could discriminate between patients with metastasis and localized controls, and the diagnostic accuracy was not significantly different for diagnosing distant metastasis in PCa (0.797, 0.722–0.872; 0.878, 0.802–0.955, respectively, *P* = 0.14, Fig. 6H, Fig. S9). Furthermore, we explored whether serum exosomal HOXD-AS1 expression is associated with prognosis of metastatic PCa patients. Survival analysis showed that high exosomal HOXD-AS1 expression PCa patients with a significantly shorter PSA recurrence-free survival survival (PRFS) and progression-free survival (PFS) (*P* = 0.006, HR = 2.05, 1.24–3.38; *P* = 0.02, HR = 2.27, 1.00–5.14, respectively (Fig. 6I, J). Additionally, univariate and multivariate analysis revealed that serum exosomal HOXD-AS1 expression together with tumor stage was prognostic factor for PRFS in PCa patients (Table 2), and an independent prognostic factor for PFS (Table S1). Collectively, these findings suggest that serum exosomal HOXD-AS1 expression correlated closely with clinical features in PCa patients, and could be applied as a potential bio-marker for diagnosing and predicting the prognosis for metastatic PCa.

**Table 1:** Association between serum exosomal HOXD-AS1 expression and clinicopathological features of prostate cancer patients.

Characteristics	Cases (%)	*χ*^*2*^	*P*-value	
**Total patients (*N*)**	130			
**Exosomal HOXD-AS1 expression**	Low	High		
**Age (year)**				
≤70	38 (29)	31 (24)	1.513	0.219
>70	27 (21)	34 (26)		
**Gleason score**				
6-7	37 (28)	28 (22)	11.377	**0.001**
8-10	28 (22)	37 (28)		
**Tumor stage**				
T2	41 (31)	30 (23)	3.755	0.053
T3-4	24 (19)	35 (27)		
**Lymphnodes status** ***N***				
Negative	59 (45)	40 (31)	15.292	**0.000**
Positive	6 (5)	25 (19)		
**Distant Metastasis M**				
M0	30 (23)	8 (6)	17.998	**0.000**
M1	35 (27)	57 (44)		

Significant *P*-values are shown in bold font.

Median serum exosomal HOXD-AS1 expression was used as cut-off value for analysis.

**Table 2:** Univariate and multivariate analysis of factors associated with PSA recurrence-free survival in metastatic prostate cancer cohort.

	Univariate			Multivariate		
Variable	HR	95% CI	*p*	HR	95% CI	*p*
Age, years (>70/≤70)	1.455	0.886–2.389	0.138			NA
Gleason score (8–10/6–7)	1.543	0.863–2.759	0.140			
Tumor stage (T3–4/T1–2)	2.381	1.430–3.967	**0.001**	2.056	1.215–3.479	**0.007**
Nodal metastasis (N1/N0)	1.361	0.796–2.326	0.260			NA
Exosomal HOXD-AS1 (high/low)	2.224	1.332–3.714	**0.002**	1.873	1.104–3.178	**0.020**

Univariate and multivariate analysis. Cox proportional hazards regression model. Variables associated with survival by univariate analyses were adopted as covariates in multivariate analyses. Significant *P*-values are shown in bold font. HR > 1, risk for death increased; HR < 1, risk for death reduced. Median relative expression of serum exosomal HOXD-AS1 was used as cut-off value for analysis.

## Discussion

Metastasis is the major cause of PCa-related death [22]. Although it has been reported that cellular communication by direct contact, hormones and metabolites in the TME participates in cancer metastasis, the significance of cellular interaction by exosomal lncRNA in PCa remains elusive. Herein, we demonstrated that a exosomal lncRNA HOXD-AS1 is involved in the metastasis of PCa. Exosomal HOXD-AS1 was internalized by cells, enhancing cellular motility by inducing metastatic phenotype in vitro and promoted distant metastasis in vivo. Mechanistically, exosomal HOXD-AS1 act as ceRNA to specifically bind with miR-361-5p, which subsequently upregulated the expression of its target FOXM1, resulting in the metastasis of PCa. Additionally, we also demonstrated that serum exosomal HOXD-AS1 could be applied as a marker for diagnosis and predicting the prognosis for metastatic PCa. These findings provided in-depth mechanistic and translational insights into the axis by which exosomal HOXD-AS1 promotes PCa metastasis, and that it may emerge as a novel marker for liquid biopsy in PCa.

Exosomes have been studied for their role in intercellular communication in the TME. Previously, several studies have revealed that exosomal lncRNAs were involved in the proliferation, therapeutic resistance, and metastasis in various cancers, and its biological effect is achieved by direct transferring RNA to the recipient cells [12, 23–25]. Herein, we found that CRPC-Exos was directly internalized into PCa cells, and promoted cell motility by transferring HOXD-AS1. Notably, in vivo study revealed that exosomal HOXD-AS1 strongly promoted bone metastasis, the most common type of distant metastasis of PCa. These results revealed the significance of of TME derived exosomal HOXD-AS1 in the metastasis of PCa.

HOXD-AS1 has been characterized as a ceRNA to modulate progression in a variety of cancers, including glioma [26], hepatocellular carcinoma [27], and cervical cancer [28]. Despite the fact that we previously reported that HOXD-AS1 is distributed both in the cytoplasm and nucleus, and nucleic HOXD-AS1 act as a molecular scaffold to mediate gene transcription [15], its function in the cytoplasm is unclear. In the present study, we identified exosomal HOXD-AS1 directly interacted with miR-361-5p, one of the most enriched miRNAs in PCa. Importantly, miR-361-5p is downregulated in CRPC and represses PCa progression by directly inhibiting its downstream target expression [17, 20]. Moreover, miR-361-5p is also a key tumor suppressor many types of cancers [18, 19], which could inhibit tumor metastasis through different mechanisms [18, 29], including repressing EMT [19, 30]. Last but not least, FOXM1, as one of the most important oncogenes in PCa as well as the direct target of miR-361-5p [19, 31], was revealed as the target of exosomal HOXD-AS1 in our current study. Therefore, our research provided that exosomal HOXD-AS1 act as a ceRNA that binding with miR-361-5p, facilitating its target FOXM1 expression therefore promoting PCa metastasis, which expanded current knowledge on HOXD-AS1 regulation in PCa.

Another important finding in the present study was that we proposed a novel aspect to support the co-existence of adaption and selection models in PCa progression and metastasis. Initially, these two models were proposed to explain the progression of PCa and thought to be mutually exclusive [2, 5, 32–34]. However, recent studies using more sophisticated techniques demonstrated that these two models co-exist and work dependently during the progression of PCa [35, 36]. Pre-existing therapeutic-resistant PCa cells are identified with unique gene expression signatures, which could be convertible during PCa progression [36–38]. In our present study, we identified that CRPC cell secreted exosome could promote the migration of PCa cells in vitro and in vivo by transmitting HOXD-AS1. Acquired exosomal HOXD-AS1 in PCa cells triggered metastatic signaling by regulating the miR-361-5p/FOXM1 axis. Our findings supported the theories that both pre-existing CRPC cells and acquired epigenetic changes could contribute to the metastasis of PCa. Common PCa cells could be converted into more aggressive types with metastatic features, in a novel pathway of intercellular communication mediated by exosomal lncRNA.

Exosomal RNAs are emerging as novel diagnostic bio-makers for its non-invasiveness and stable in body fluids [39]. Exosomal androgen receptor splice variant 7 (*AR-V7*) detection has been applied clinically as the marker to predict the sensitivity of novel anti-androgen regimens [40]. Moreover, exosomal microRNAs and lncRNAs are also reported as useful markers for diagnosing PCa [41–43]. Herein, we found that HOXD-AS1 was overexpressed in serum exosomes from patients with metastatic PCa, and it was positively associated with nodal and distant metastasis. Importantly, the efficacy of serum exosomal HOXD-AS1 as bio-marker for metastatic PCa diagnosis and prognosis was evaluated. We also evaluated the diagnostic efficacy of PSA in PCa metastasis based on our cohort. Although the ROC result was not statistically significant different from that of serum exosomal HOXD-AS1, PSA still showed moderate advantage. However, detection of exosomal HOXD-AS1 is still meaningful for its diagnostic value on metastatic burden and predicting prognosis, which is of great importance in clinical decision making [44–46]. As a result, exosomal HOXD-AS1 analysis could be utilized for detection of metastasis and predicting the prognosis of metastatic PCa patients.

In summary, our findings revealed evidence of the mechanism in which CRPC cell-derived exosomal HOXD-AS1 promoted PCa metastasis by modulating miR-361-5p/FOXM1 axis. We also reported that serum exosomal HOXD-AS1 detection could be applied as a marker for metastatic disease, as well as predicting the prognosis of PCa patients. Our study not only identifies a crucial mechanism of exosomal lncRNA-mediated intercellular communication from CRPC cells to the TME, which endowed common PCa cell with metastatic features, but also develops a potential non-invasive diagnostic approach for PCa.

## Material and methods

### Cell culture

The cell lines used in this study were the human prostate cancer cells LNCaP and PC-3 (ATCC, Manassas, VA, USA), and the CRPC cell line LNCaP-Bic and LNCaP-AI as previously reported [15, 47]. And the cells were cultured as previously described by us [15].

### Human tissue and serum samples

A total of 36 and 9 cases paraffin embedded PCa and benign prostate hypertrophy (BPH) tissues were obtained by surgery or needle biopsy, and 130 cases of serum samples were collected from treatment-naïve patients after their initial pathological diagnosis from the 1^st^ Affiliated Hospital of Kunming Medical University. All the samples were pathologically diagnosed as prostate adenocarcinoma by two pathologists. The clinical features of the patients are listed in Table 1. The high volume and low volume in the metastatic patients were characterized according to the standard described in the CHAARTED trial [48]. All experiments were conducted with the approval of the Committees for Ethical Review of Research involving Human Subjects at the 1^st^ Affiliated Hospital of Kunming Medical University. Informed consent was obtained from all participants prior to sample collection.

### RNA extraction and real-time quantitative PCR (qPCR) analysis

The experiments was conducted as previously described [49]. The relative gene expression was calculated using the 2^-∆∆Ct^ method. The transcription level of GAPDH was used as an internal control for mRNAs, and U6 was used as control for miR-361-5p. All specific method and primers are listed in Supplementary materials and methods and Table S2.

### Plasmid and miRNA transfection and lentivirus transduction

The pCDNA3.1-HOXD-AS1 overexpressing vector was constructed, and stable knockdown of HOXD-AS1 in PCa cells were obtained from our previous report [15]. The human hsa-miR-361-5p mimics was synthetized by GenePharma (GenePharma, Suzhou, China). The oligos used in knockdown and miRNA transfection was listed in Table S3. The different segment of HOXD-AS1 was PCR-amplified from pCDNA3.1-HOXD-AS1 vector and cloned into psiCHECK2 luciferase vector (Promega, Madison, WI USA). The list of primers used in cloning reactions is presented in Table S4. Transfection of miRNA and plasmids was performed using Lipofectamine 3000 (Thermo Scientific, Waltham, MA USA).

### Animal study

All mouse experiments were approved by the Institution of Animal Care and Use Committee of Kunming Medical University (approval No. KMMU2020213) and housed as previously reported [50]. For the bone metastasis study, PC-3-luc cells were pre-incubated with either PBS or CRPC-Exos at a concentration 10 μg/ml in medium supplemented with exosome-depleted FBS for 48 h (each group *n* = 10, estimated 40–50% proportion of metastasis from preliminary experiment.). BALB/c-nu mice (4-week old, 18–20 g) were anesthetized and randomly divided into 4 groups, the pre-treated cells were slowly inoculated into the left cardiac ventricle at 5 × 10^5^ cells in 100 μL of PBS per mice. Then either PBS or 20 μg indicated exosomes at 50 μl volume were injected intra-cardiac under anesthesia weekly at the same time bioluminescence imaging was conducted. Osteolytic lesions were identified on radiographs as radiolucent lesions in the bone. Each bone metastasis was scored as previously reported [51, 52] based on the following criteria: 0, no metastasis; 1, bone lesion <1/4 of the bone width; 2, bone lesion involving 1/4 to 1/2 of the bone width; 3, bone lesion over 1/2 to 3/4 of the bone width; and 4, bone lesion >3/4 of the bone width. The bone metastasis score for each mouse was the sum of the scores of all bone lesions from four limbs. For survival studies, mice were monitored daily for signs of discomfort and were either euthanized all at one time or individually when presenting signs of distress, such as a 10% loss of body weight, or paralysis. For immunohistochemistry (IHC) of bone sections, anti-firefly luciferase antibody (ab185924, 1:250, Abcam, Massachusetts, USA) was used and conducted as previously reported [49].

### Other materials and methods

RNA in situ hybridization, isolation of exosomes, transmission electron microscopy (TEM), nanoparticle tracking analysis, exosomes tracking, Western Blot and antibody information, in vitro assays, and other methods are described in Supplementary materials and methods.

## Supplementary information

Author Contribution Form

Figure S1

Figure S2

Figure S3

Figure S4

Figure S5

Figure S6

Figure S7

Figure S8

Figure S9

Table S1

Table S2

Table S3

Table S4

Supplementary Materials and Methods

Supplementary Figure Legends

Supplementary References

checklist

Author Contribution statement

## Data Availability

The primary data from microarray analysis have been deposited to the Gene Expression Omnibus and the accession numbers is GSE93929. The rest of the data used and analyzed during the current study are available from the corresponding author on reasonable request.
