# Supplementary material for: Exosomal long noncoding RNA HOXD-AS1 promotes prostate cancer metastasis via miR-361-5p/FOXM1 axis
Source: Cell Death Dis. 2021 Dec 4;12(12):1129. doi: 10.1038/s41419-021-04421-0 (PMC8643358; doi:10.1038/s41419-021-04421-0)
Supplement: Supplementary file 1 — Author Contribution Form [file 41419_2021_4421_MOESM1_ESM.pdf]

DECLARATION OF CONTRIBUTIONS TO ARTICLE

ADMC

Manuscript Number:	Journal Name:	
CDDIS-21-2927	*Cell Death & Disease*	(the 'Journal')

Proposed Title of the Contribution:

P.G. and X.L. conceptualized the study, acquired funding support, and revised the final (the 'Contribution)

Author(s):

Yongming Jiang, Hui Zhao , Yuxiao Chen, Kangjian Li, Tianjie Li, Jianheng Chen, (the 'Authors')

For all *CDDis* articles, each person named as an author in the published version must be able to show he or she has contributed substantially to the article.

Authorship credit should be based on 1) substantial contributions to conception and design, acquisition of data, or analysis and interpretation of data; 2) drafting the article or revising it critically for important intellectual content; and 3) final approval of the version to be published. Authors should meet conditions 1, 2 and 3.

Any person who cannot be shown to have made a substantial contribution to the article cannot be listed as an author in the final version. The name of any person who is deemed to have made a minor contribution can, however, appear in the Acknowledgments section of the article.

Please complete the table below to indicate the contributions of all named authors to the manuscript.

Author Full Name:	Specification of Contribution to the Manuscript:
Yongming Jiang	in vitro and in vivo experimental, construction of vectors,
Hui Zhao	in vitro and in vivo experimental, construction of vectors,
Yuxiao Chen	molecular experiment including qPCR and western blot
Kangjian Li	molecular experiment including qPCR and western blot,isolated
Tianjie Li	isolated and characterized the exosomes
Jianheng Chen	molecular experiment including qPCR and western blot
Baiyu Zhang	luciferase assays, RIP, and RNA ISH
Caifen Guo	luciferase assays, RIP, and RNA ISH
Liangliang Qing	luciferase assays, RIP, and RNA ISH
Jihong Shen	collected clinical samples and profiles, and finished the follow-up
Xiaodong Liu	conceptualized the study, acquired funding support, and revised
Peng Gu	luciferase assays, RIP, and RNA ISH, conceptualized the study,

Please complete the table below to indicate the contributions of all named authors to the figures.

Figure 1:

A-B: Yongming Jiang generated the data and graph, C-D: Yuxiao Chen and Tianjie Li generated the data and graph, E-G: Baiyu Zhang and Caifen Guo generated the data and labeled images, Jihong Shen collected clinical samples, H: Yongming Jiang did the correlation analysis. Hui Zhao assembled the figure.

Figure 2:

A-C: Tianjie Li, Kangjian Li, Huizhao and Yuxiao Chen enriched the exosomes and generated the data and graph, D-I: Yongming Jiang and Hui Zhao generated the data and graph, J-K: Yuxiao Chen and Jianheng Chen generated the data. Hui Zhao assembled the figure.

Figure 3:

A: Hui Zhao and Tianjie Li performed the experiment, captured and labled images, B-C: Yongming Jiang and Caifen Guo generated the data and graph, D-G: Yongming Jiang and Hui Zhao did the experiment, captured the images and generated the graphs. Yongming Jiang assembled the figure.

Figure 4:

A-E: Yongming Jiang and Hui Zhao performed the animal study and generated the images and graphs, F: Baiyu Zhang and Liangliang Qing did the staining of tissue, captured and labled the images. Hui Zhao assembled the figure.

Figure 5:

A: Hui Zhao analyzed and visualized the bioinformatic analysis, B-C: Yongming Jiang and Yuxiao Chen generated the data and graph, D-G: Hui Zhao and Yongming Jiang constrcted the vectors, and Baiyu Zhang did the luciferase assays, Caifen Guo generated the graphs, H-J: Peng Gu and Yuxiao Chen generated the data and graphs, K-J: Kangjian Li, Yuxiao Chen and Caifen Guo generated the data. Yongming Jiang assembled the figure.

Figure 6:

A-C: Tianjie Li, Kangjian Li and Caifen Guo enriched the exosomes and generated the data and graphs. D-J: Jihong Shen collected the serum samples, Jihong Shen and Xiaodong Liu coolected the clinical information and analyzed it, Yongming Jiang finished the follow-up information, Yuxiao Chen and Hui Zhao deteced the expression by qPCR and generated the graphs. Hui Zhao assembled the figure.

Signed for and on behalf of the Author(s):	Print Name:	Date:
顾鹏	Peng Gu 顾鹏	2021-7-25
