# Supplementary figures and images for: Exosomal long noncoding RNA HOXD-AS1 promotes prostate cancer metastasis via miR-361-5p/FOXM1 axis
Source: Cell Death Dis. 2021 Dec 4;12(12):1129. doi: 10.1038/s41419-021-04421-0 (PMC8643358; doi:10.1038/s41419-021-04421-0)

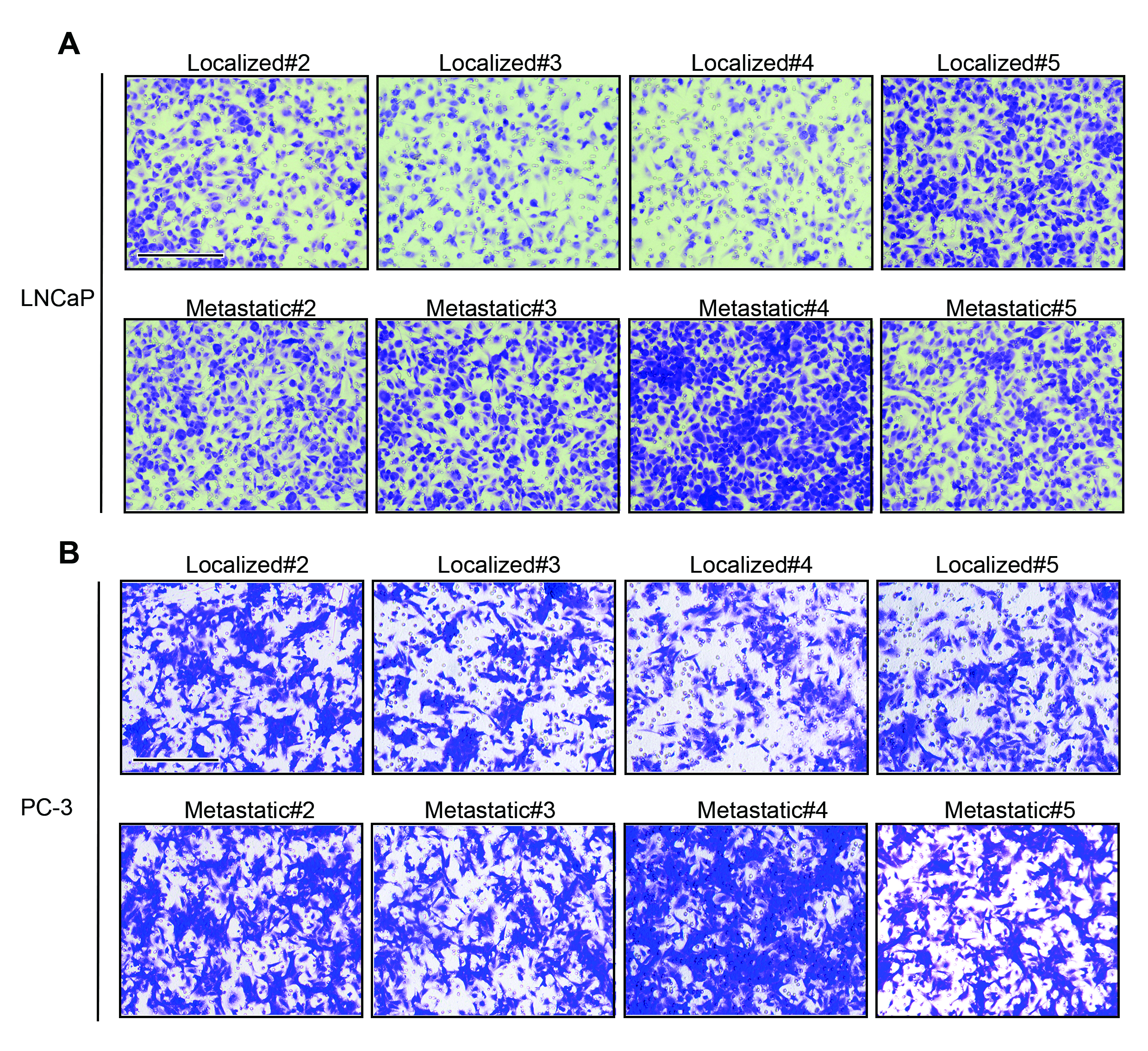

Supplement: Supplementary file 2 — Figure S1 [file 41419_2021_4421_MOESM2_ESM.tif]

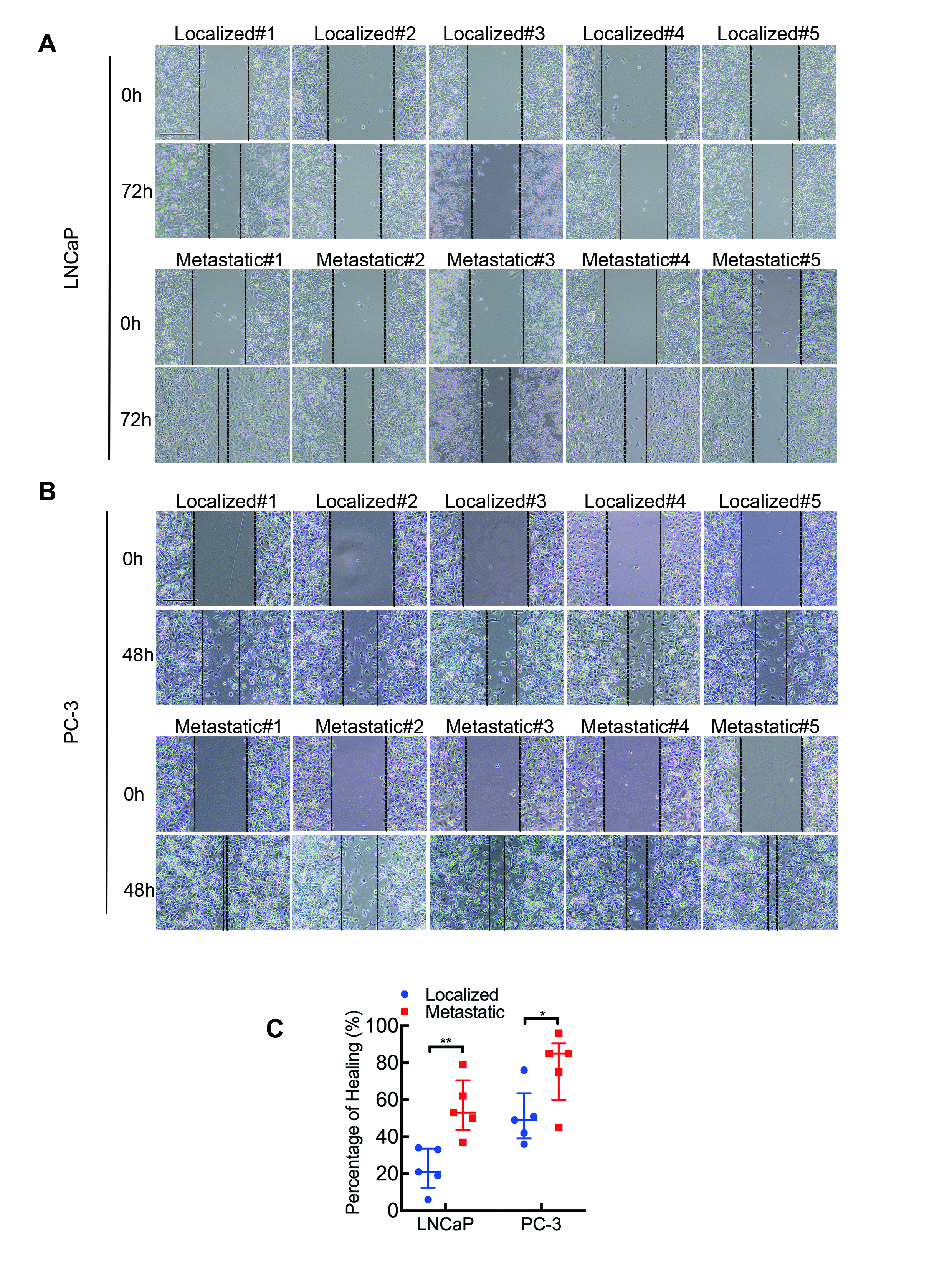

Supplement: Supplementary file 3 — Figure S2 [file 41419_2021_4421_MOESM3_ESM.tif]

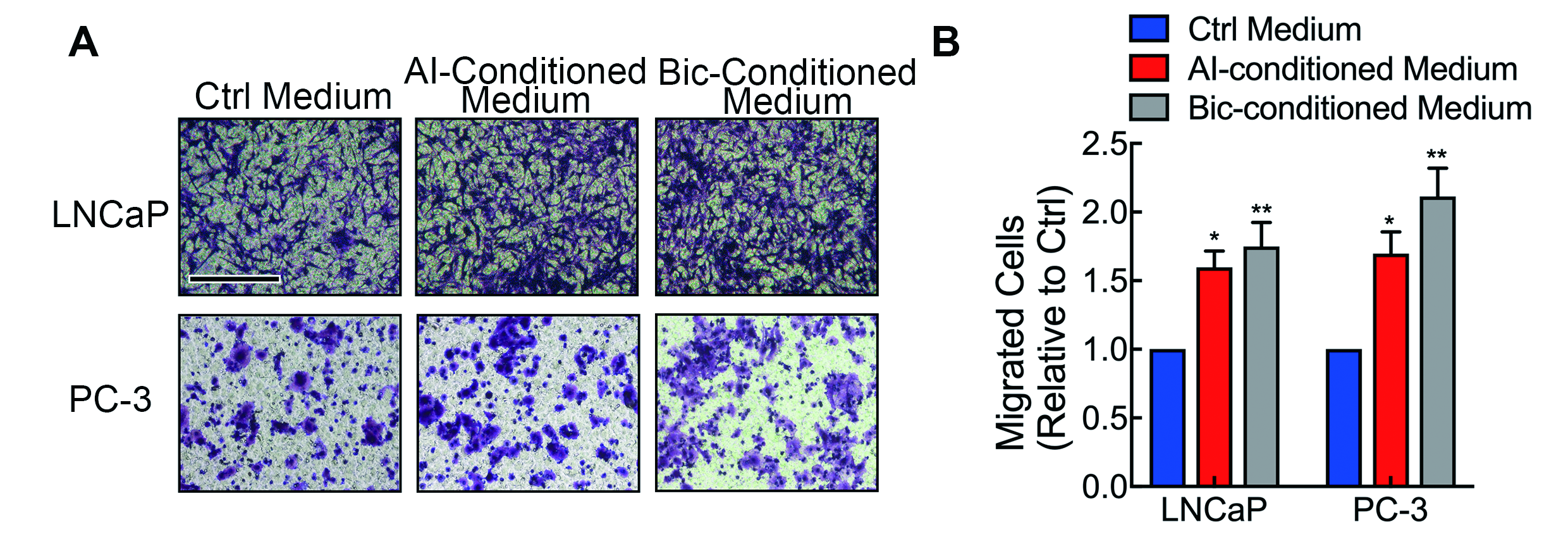

Supplement: Supplementary file 4 — Figure S3 [file 41419_2021_4421_MOESM4_ESM.tif]

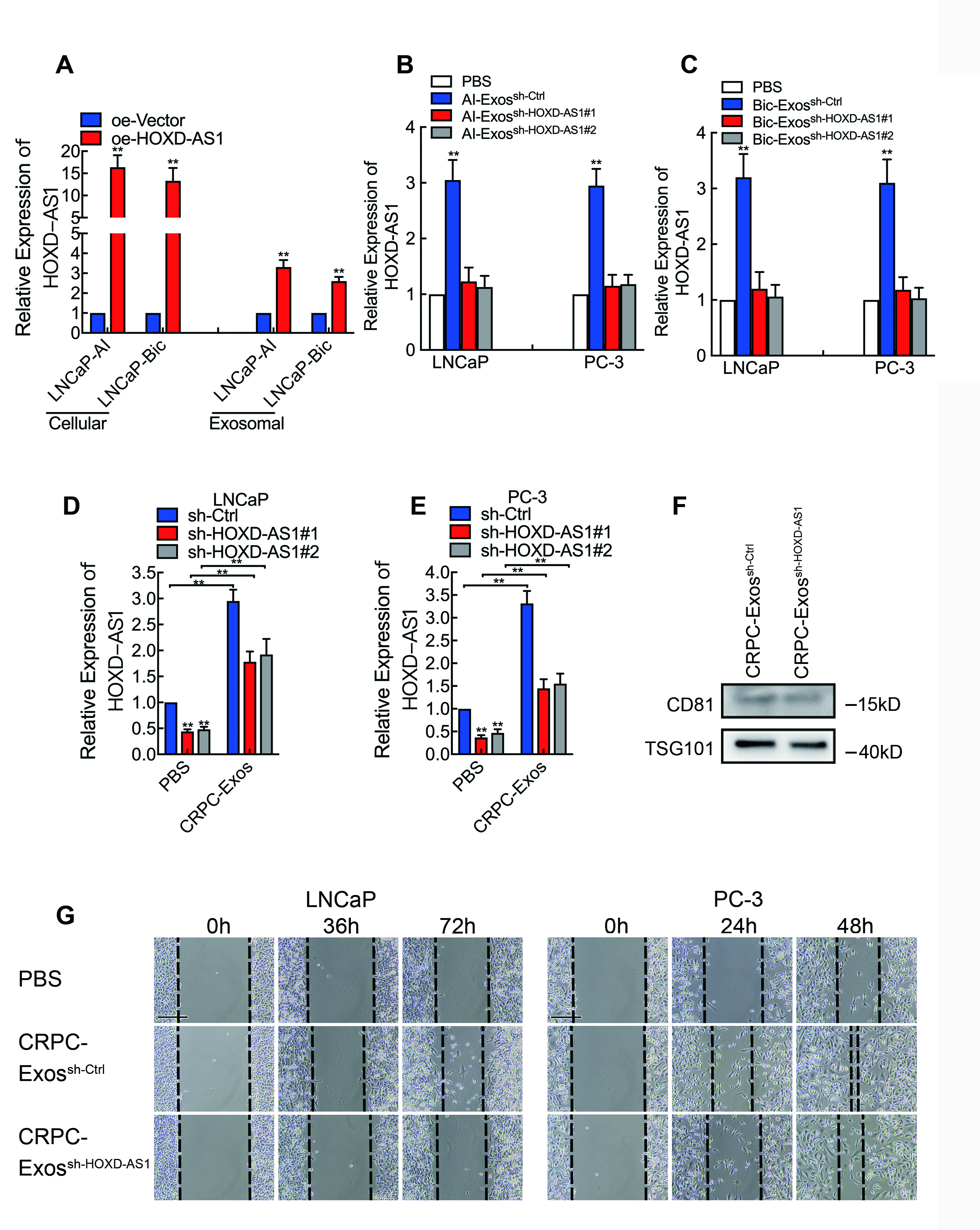

Supplement: Supplementary file 5 — Figure S4 [file 41419_2021_4421_MOESM5_ESM.tif]

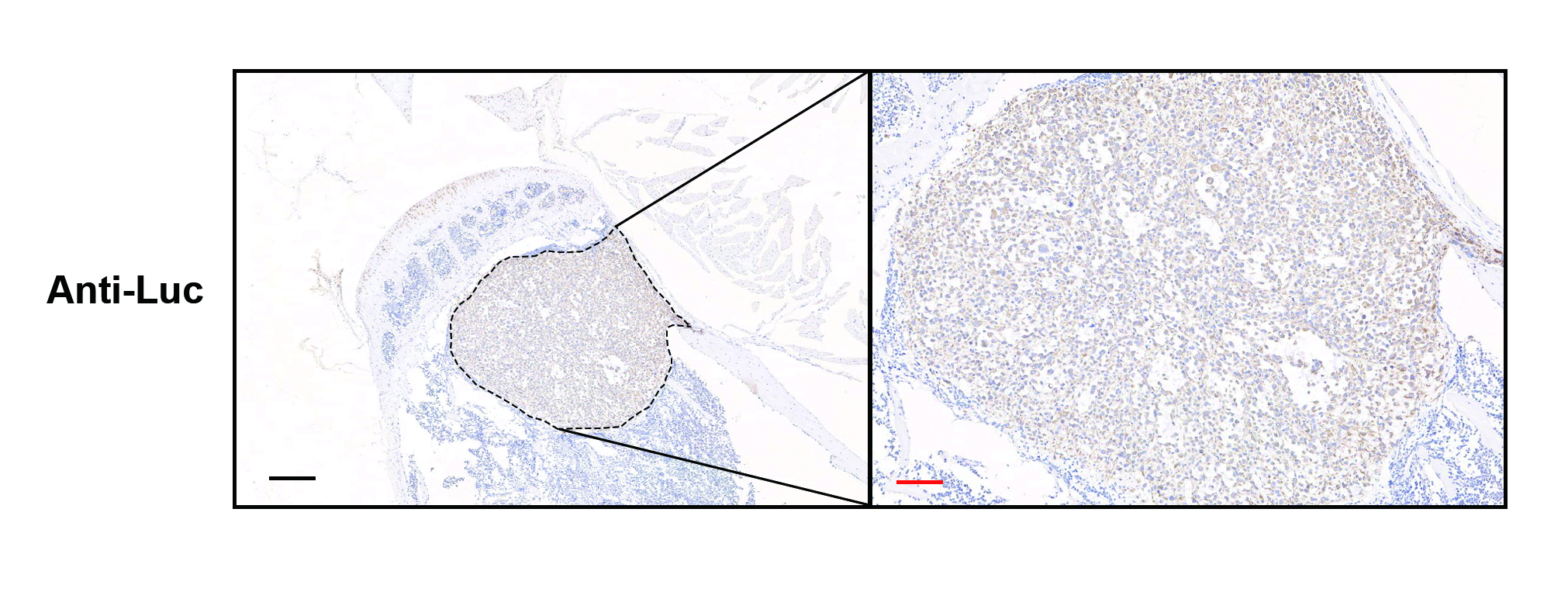

Supplement: Supplementary file 6 — Figure S5 [file 41419_2021_4421_MOESM6_ESM.tif]

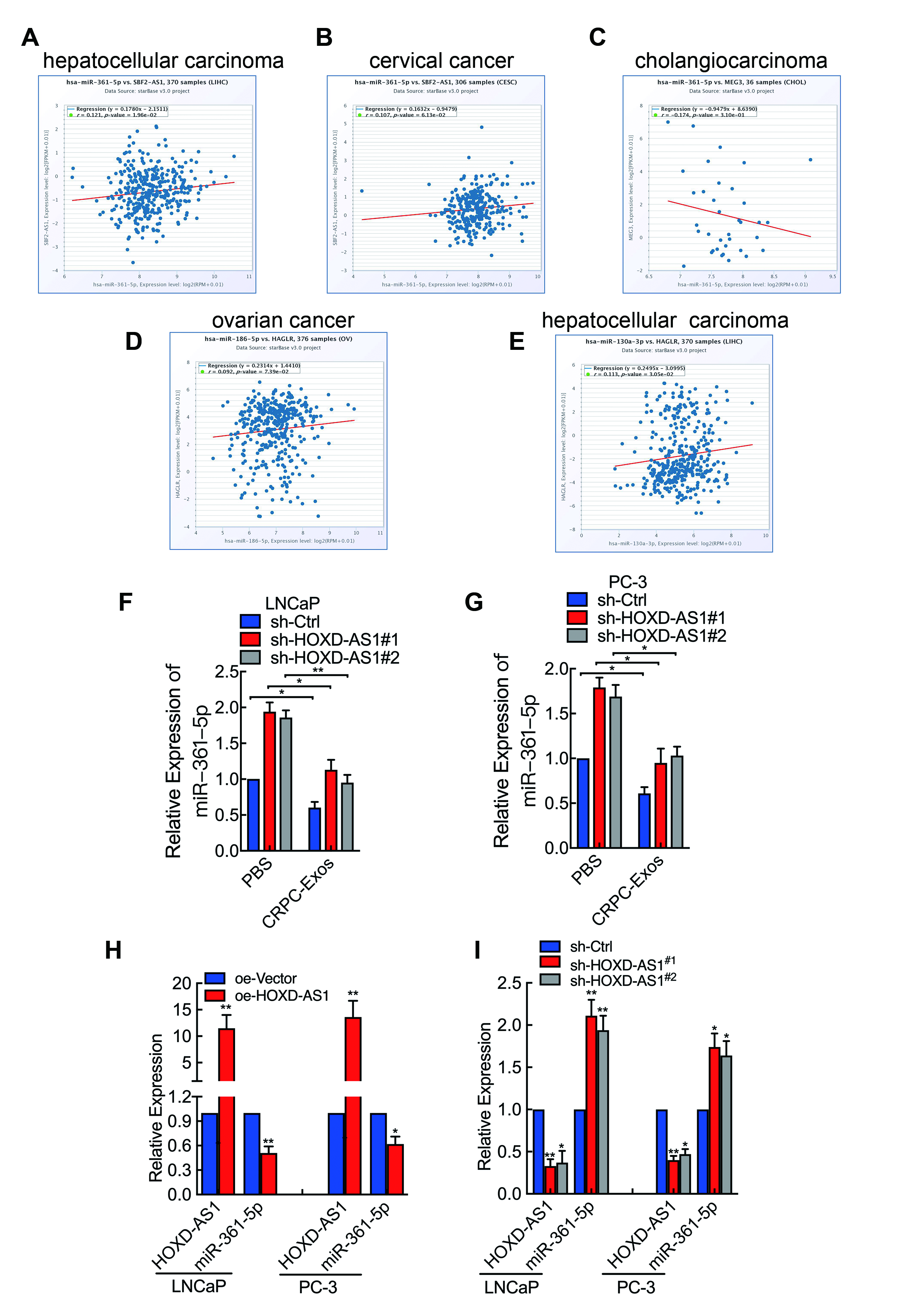

Supplement: Supplementary file 7 — Figure S6 [file 41419_2021_4421_MOESM7_ESM.tif]

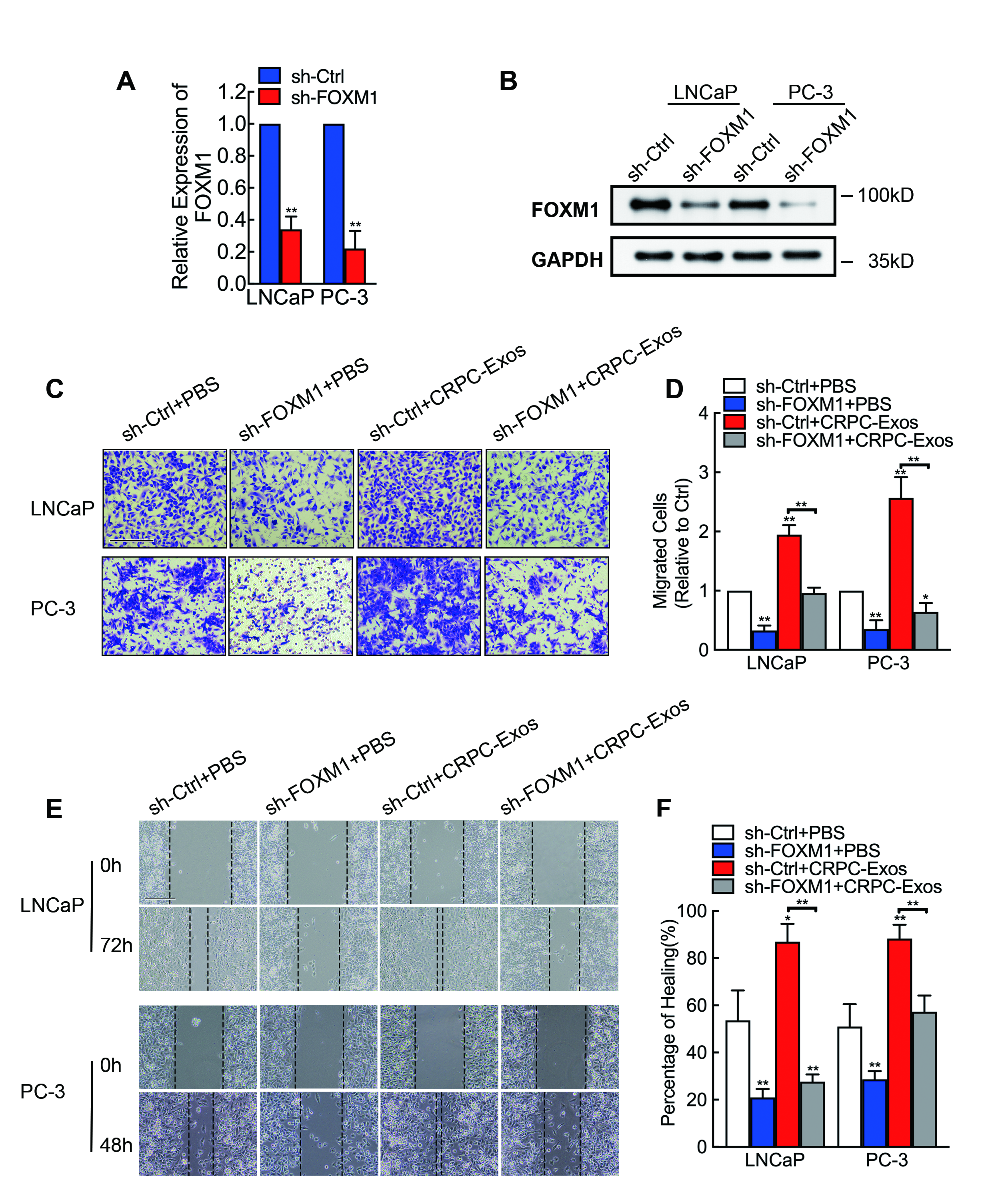

Supplement: Supplementary file 8 — Figure S7 [file 41419_2021_4421_MOESM8_ESM.tif]

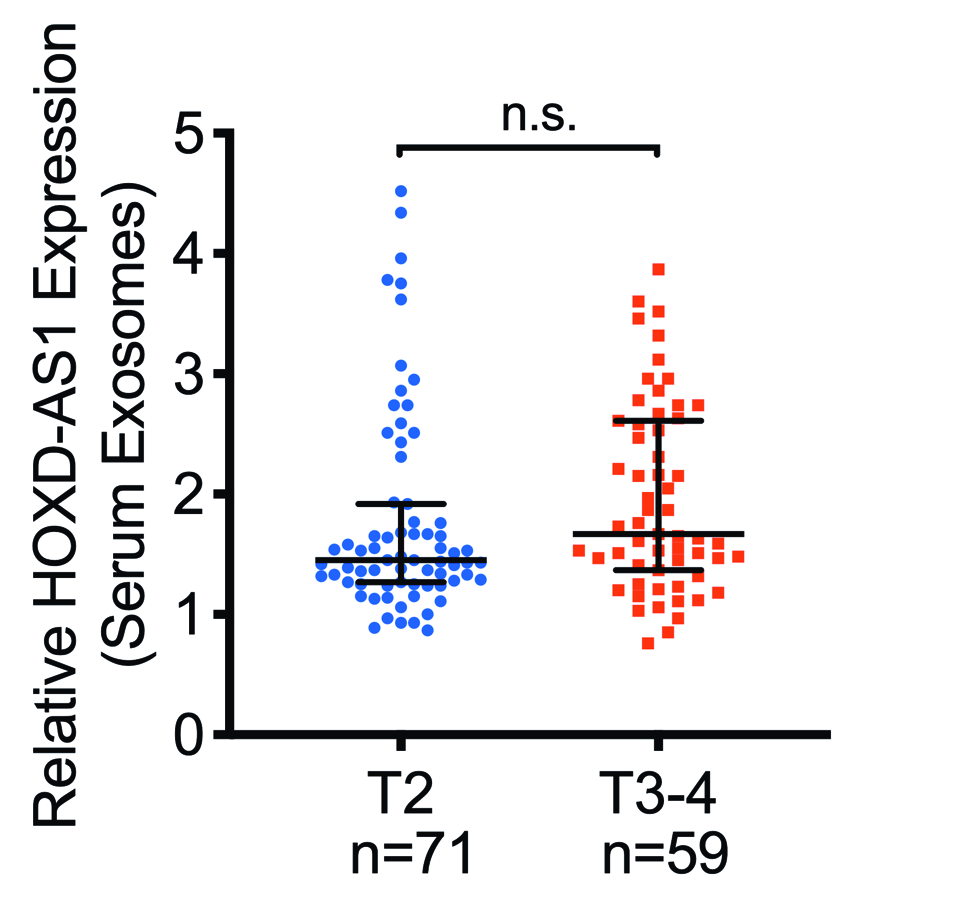

Supplement: Supplementary file 9 — Figure S8 [file 41419_2021_4421_MOESM9_ESM.tif]

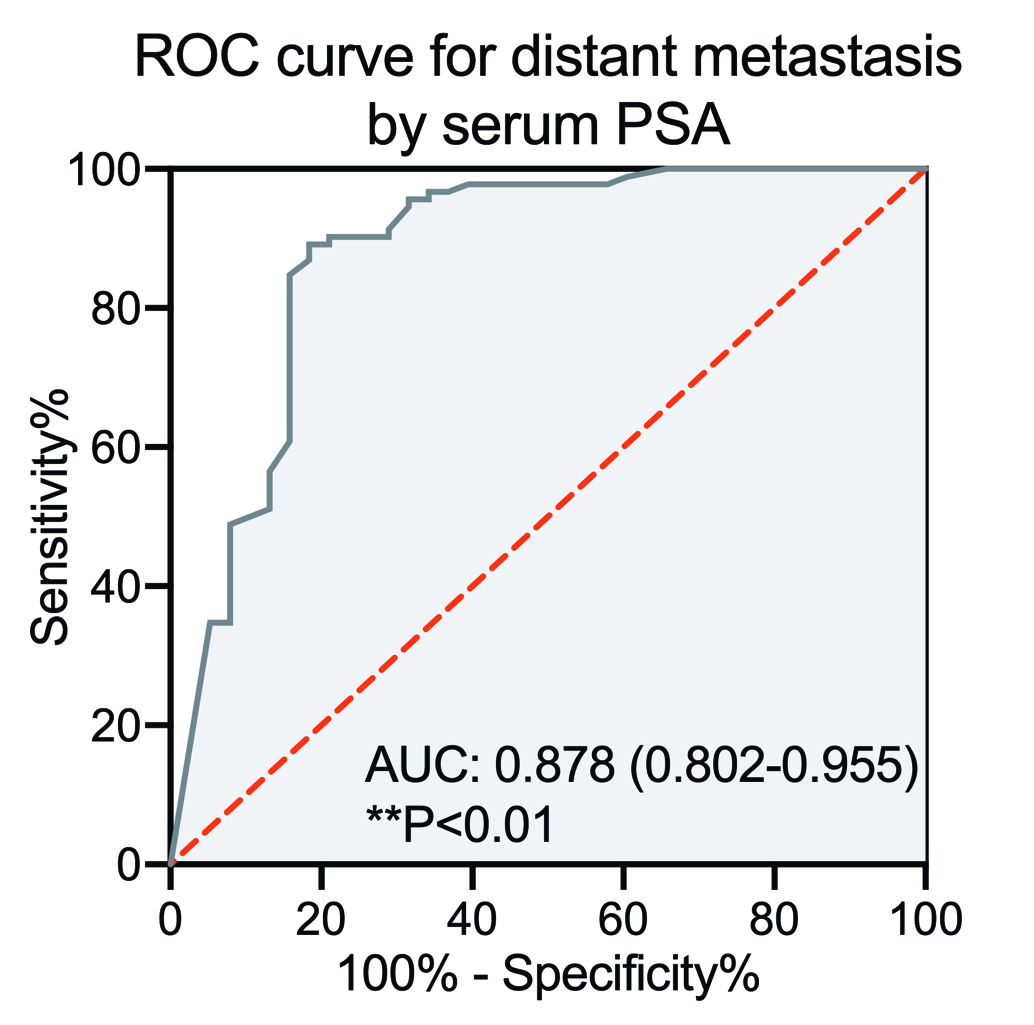

Supplement: Supplementary file 10 — Figure S9 [file 41419_2021_4421_MOESM10_ESM.tif]
