# Supplementary material for: Exosomal long noncoding RNA HOXD-AS1 promotes prostate cancer metastasis via miR-361-5p/FOXM1 axis
Source: Cell Death Dis. 2021 Dec 4;12(12):1129. doi: 10.1038/s41419-021-04421-0 (PMC8643358; doi:10.1038/s41419-021-04421-0)
Supplement: Supplementary file 11 — Table S1 [file 41419_2021_4421_MOESM11_ESM.docx]

**Supplementary Table 1**

Table S1. Univariate and multivariate analysis of factors associated with progression free survival in metastatic prostate cancer cohort.

|  | | Univariate | | |  | Multivariate | | |
| --- | --- | --- | --- | --- | --- | --- | --- | --- |
| Variable | | HR | 95% CI | *p* |  | HR | 95% CI | *p* |
| Age, years (>70/≤70) | | 1.712 | 0.774–3.787 | 0.184 |  |  |  | NA |
| Gleason Score (8-10/6-7) | | 2.055 | 0.768-5.497 | 0.151 |  |  |  | NA |
| Tumor stage (T3-4/T1-2) | | 1.998 | 0.893-4.470 | 0.092 |  |  |  | NA |
| Nodal metastasis (N1/N0) | | 1.873 | 0.814-4.308 | 0.140 |  |  |  | NA |
| Exosomal HOXD-AS1 (high/low) | | 2.380 | 1.054-5.371 | **0.037** |  |  |  | NA |
|  | Univariate and multivariate analysis. Cox proportional hazards regression model. Variables associated with survival by univariate analyses were adopted as covariates in multivariate analyses. Significant P-values are shown in bold font. HR > 1, risk for death increased; HR < 1, risk for death reduced. Median relative expression of serum exosomal HOXD-AS1 was used as cut-off value for analysis. | | | | | | | |
