# Supplementary material for: Exosomal long noncoding RNA HOXD-AS1 promotes prostate cancer metastasis via miR-361-5p/FOXM1 axis
Source: Cell Death Dis. 2021 Dec 4;12(12):1129. doi: 10.1038/s41419-021-04421-0 (PMC8643358; doi:10.1038/s41419-021-04421-0)
Supplement: Supplementary file 12 — Table S2 [file 41419_2021_4421_MOESM12_ESM.docx]

**Supplementary Table 2**

**Table S2.** The primers used in real time qPCR are listed as follows.

| Primer Name | Sequence 5’-3’ |
| --- | --- |
| HOXD-AS1 Forward | ACCTGCCTCTACTACTGCAAA |
| HOXD-AS1 Reverse | GCAAAGACAATATAAGGGCCC |
| U6 Forward | CTCGCTTCGGCAGCACATATAC |
| U6 Reverse | AACGCTTCACGAATTTGCGTGTC |
| E-Cadherin Forward | TTCCCTACGTATACCCTGGTG |
| E-Cadherin Reverse | CGGAGGATTATCGTTGGTGTC |
| Vimentin Forward | AGTCCACTGAGTACCGGAGAC |
| Vimentin Reverse | CATTTCACGCATCTGGCGTTC |
| hsa-miR-361-5p Forward | TTATCAGAATCTCCAGGGGTAC |
| miR-Reverse | Provided with the Mir-X miRNA qRT-PCR TB Green Kit |
| GAPDH Forward | CAAGGCTGAGAACGGGAAG |
| GAPDH Reverse | TGAAGACGCCAGTGGACTC |
