# Supplementary material for: Exosomal long noncoding RNA HOXD-AS1 promotes prostate cancer metastasis via miR-361-5p/FOXM1 axis
Source: Cell Death Dis. 2021 Dec 4;12(12):1129. doi: 10.1038/s41419-021-04421-0 (PMC8643358; doi:10.1038/s41419-021-04421-0)
Supplement: Supplementary file 13 — Table S3 [file 41419_2021_4421_MOESM13_ESM.docx]

**Supplementary Table 3**

**Table S3.** The sequences of oligos used in the experiments are listed as follows.

| Oligo Name | Sequence 5’-3’ |
| --- | --- |
| HOXD-AS1 sh#1 | GAAAGAAGGACCAAAGTAATG |
| HOXD-AS1 sh#2 | GCCCTTTCTGACCTGCTTATG |
| sh-FOXM1 | GGCTGCACTATCAACAATAGC |
| sh-Control | CCTAAGGTTAAGTCGCCCTCG |
| hsa-miR-361-5p mimics | UUAUCAGAAUCUCCAGGGGUAC |
| Control mimics | UUGUACUACACAAAAGUACUG |
