# Supplementary material for: Exosomal long noncoding RNA HOXD-AS1 promotes prostate cancer metastasis via miR-361-5p/FOXM1 axis
Source: Cell Death Dis. 2021 Dec 4;12(12):1129. doi: 10.1038/s41419-021-04421-0 (PMC8643358; doi:10.1038/s41419-021-04421-0)
Supplement: Supplementary file 14 — Table S4 [file 41419_2021_4421_MOESM14_ESM.docx]

**Supplementary Table 4**

**Table S4.** The primers used in gene clone are listed as follows.

| Primer Name | Sequence 5’-3’ |
| --- | --- |
| HOXD-AS1(1-1161) Forward | GCGATCGCAAAGCAGCCCAGGGCCGCCGCCCA |
| HOXD-AS1(1-1161) Reverse | GCGGCCGCCACCTTATCCAGGGACCTGACAGA |
| HOXD-AS1(1161-2400) Forward | GCGATCGCGAGGTCTGAAAGCCAGGAGACAGA |
| HOXD-AS1(1161-2400) Reverse | GCGGCCGCGATCTGTTCCTGCCATTGGCGTGA |
| HOXD-AS1(2400-3638) Forward | GCGATCGCCGAGCTATTAGAAGTCTCGGGAAC |
| HOXD-AS1(2400-3638) Reverse | GCGGCCGCCCATCATTTGGAGACGCGTACCTA |
| HOXD-AS1(3638-4148) Forward | GCGATCGCTTTCTTTTTCTACACCTTAGCTTT |
| HOXD-AS1(3638-4148) Reverse | GCGGCCGCTCATTTCTCAAAAACCACAGTCCT |
| HOXD-AS1(1-301) Forward | GCGATCGCAAAGCAGCCCAGGGCCGCCGCCCA |
| HOXD-AS1(1-301) Reverse | GCGGCCGCGGAGGTCTTGTTGGGCCAGGAATG |
| HOXD-AS1(301-600) Forward | GCGATCGCCTGGCCAGGTTTTGAGCCCTGCTT |
| HOXD-AS1(301-600) Reverse | GCGGCCGCATTAGCTGAAACTTCCCTCTGAAG |
| HOXD-AS1(600-900) Forward | GCGATCGCTTCTCAGGAAAAGGTCAAAACAGG |
| HOXD-AS1(600-900) Reverse | GCGGCCGCGATTACCTATACTAGCAGCCTTTG |
| HOXD-AS1(900-1161) Forward | GCGATCGCCACTATCTTTTAATACTCCTAACA |
| HOXD-AS1(900-1161) Reverse | GCGGCCGCCACCTTATCCAGGGACCTGACAGA |
| HOXD-AS1(900-1161) Forward-mut | GCGATCGCCACTATCTTTTAATACTCCTAACA |
| HOXD-AS1(900-1161) Reverse-mut | GCGGCCGCCACTATCTTTTAATACTCCTAACA |
