# Supplementary material for: Exosomal long noncoding RNA HOXD-AS1 promotes prostate cancer metastasis via miR-361-5p/FOXM1 axis
Source: Cell Death Dis. 2021 Dec 4;12(12):1129. doi: 10.1038/s41419-021-04421-0 (PMC8643358; doi:10.1038/s41419-021-04421-0)
Supplement: Supplementary file 15 — Supplementary Materials and Methods [file 41419_2021_4421_MOESM15_ESM.docx]

**Supplementary Materials and Methods**

***In situ* hybridization (ISH)**

HOXD-AS1 expression was examined using ISH in formalin-fixed, paraffin-embedded (FFPE) samples, as previously described by us^6^. The 5’-and 3’-DIG labeled HOXD-AS1 probe was synthesized by Sangon (Shanghai, China). The sequence was 5’- CGCATCTCTATTTGGTTTGA -3’. The staining intensity of ISH was graded as follows: 0 (no staining), 1 (weak staining, light brown), 2 (moderate staining, brown) and 3 (strong staining, deep brown). The intensity of staining was multiplied by the percentage of positive cells (0%-100%), and the H-score (0-300) of each tissue was obtained for statistical analysis. The score of ISH in the FFPE samples was blindly quantified by two pathologists and the average H-score (0-300) of each tissue was obtained for statistical analysis.

**Isolation of cell derived exosomes and serum exosomes**

LNCaP-AI and LNCaP-Bic cells were cultured for 48 hours in phenol-red free RPMI-1640 medium (Thermo Scientific, Waltham, MA USA) without supplements. Exosomes were isolated from the supernatant of CRPC cells by differential centrifugations as previously described^7^. Breifly, the medium was harvested and centrifuged at 300g for 10 min at 4℃. The supernatant was further centrifuged at 16500g for 20 min at 4℃ and filtered through a 0.22μm filter. Exosomes were then pelleted by ultracentrifugation at 120,000g for 70 min at 4℃. Exosome pellets were resuspended in 0.22 μm-filtered PBS for subsequent experiments or stored at -80℃.

Exosomes were isolated from the human serum samples by Total Exosome Isolation kit (for serum) according to the manufacturer’s instructions (Thermo Scientific, Waltham, MA USA). Briefly, serum samples were centrifuged at 2000g for 30 min at 4℃, then 200μl serum samples were mixed with 40μl Total Exosome Isolation reagent and incubated at 4℃ for 1 hour. The mixtures were centrifuged at 10000g for 30 min at room temperature. Exosome pellets were resuspended in 50μl 0.2 μm-filtered PBS.

Before using exosomes either isolated from medium or serum, exosomes were quantified based on protein concentration with micro BCA Protein Assay Kit (Thermo Scientific, Waltham, MA USA). Then identical amount of exosomes were applied to subsequent experiment. For *in vitro* assays, exosomes were diluted to a concentration of 10μg/mL for indicated treatment. In the manuscript, LNCaP-AI derived exosomes were used to represent CRPC cell secreted exosomes and mentioned as CRPC-exos.

**Transmission electron microscopy**

Exosomes were adsorbed to a 400-mesh carbon-coated copper grids and stained with phosphotungstic acid. Morphologies of the samples were observed by a JEOL JEM-100SX transmission electron microscope (JEOL Ltd., Tokyo, Japan).

**Nanoparticle tracking analysis**

The number and size of the exosomes were directly tracked using the NS300 instrument (Malvern Instruments Ltd., Worcestershire, UK). The exosome pellets were resuspended and diluted in PBS to obtain a concentration within the recommended range (1×10^7^~1×10^9^ particles/mL). The samples were loaded into the sample chamber at ambient temperature. One 60s video was acquired for each sample. The videos were then analyzed with the NTA3.2 software, which identified and tracked each particle's center under Brownian motion to measure the average distance the particles moved on a frame-by-frame basis.

**Exosomes Tracking**

Exosomes were stained with PKH67 Green Fluorescent Cell Linker Kit (Sigma Aldrich, St Louis, USA) according to the manufacturer’s instruction. PKH67-labeled exosomes were collected by ultracentrifugation and resuspended in medium containing 10% exosome-depleted FBS. Then exosomes were added to PCa cells and incubated for 24 hours. Then cells were fixed with 4% formaldehyde after washing twice with PBS. The nuclei were stained with DAPI. A Zeiss confocal microscope was used to obtain the images.

**RNA extraction and real-time quantitative PCR (qPCR) analysis**

Total RNA from cells and exosomes was extracted using the TRIzol reagent (Thermo Scientific, Waltham, MA USA). RNA quantity was measured using a NanoDrop 2000 spectrophotometer (Thermo Scientific, Waltham, MA USA). Total RNA was reverse transcribed by PrimeScript RT Master Mix (Takara Biotechnology Co., Ltd., Dalian, China). For miRNA reverse-transcription, Mir-X miRNA First-Strand Synthesis Kit (Takara Biotechnology Co., Ltd., Dalian, China) was used according to the manufacture’s protocol. qRT-PCR analysis was conducted using the TBGreen II (Takara Biotechnology Co., Ltd.) and Mir-X miRNA qRT-PCR TB Green Kit (Takara Biotechnology Co., Ltd.), and analyzed on a Roche Light-Cycler 480 system (Roche, CA, USA). The relative gene expression was calculated using the 2^-∆∆Ct^ method. The transcription level of GAPDH was used as an internal control. All specific primers are listed in Table S2.

***In vitro* cell migration and wound-healing assays**

LNCaP and PC-3 were treated with CRPC-exos at 10 μg/ml in the functional assays. Transwell and Wound-healing assays were performed to detect cell migration. The details were described in our previous study^8, 9^.

**Western Blot and antibodies**

Western blotting was performed as previously described^10, 11^. Primary antibodies specific to E-Cadherin, Vimentin, GAPDH (#9728, #97166, 1:1000, Cell Signaling Technology, MA, USA), TSG101, CD81, FOXM1 (ab125011, ab109201, ab207298, 1:1000, Abcam, Massachusetts, USA) were used. The blots were then incubated with goat anti-rabbit or anti-mouse secondary antibody (Cell Signaling Technology, MA, USA) and visualized using enhanced chemiluminescence.

**Luciferase Assay**

5×10^4^ cells were seeded in triplicate in 24-well plates and cultured for 24 hours and performed as previously described^6^. 250ng luciferase vectors and miRNA mimics at final concentration of 50 nmol/L were co-transfected by lipofectamine 3000. Luciferase and Renilla signals were measured 36 hours after transfection using the Dual Luciferase Reporter Assay Kit (Promega, Madison, WI USA) according to the manufacturer’s protocol.

**RIP assay**

The RIP was performed as we described previously^12^ using the EZ-Magna RIP kit (Millipore Massachusetts, USA). Ago2 and HA antibody (1:200, Abcam, Massachusetts, USA) were used. Normal rabbit IgG (provided with the kit) was used as a negative control.

**Statistical analysis**

Quantitative data were presented as the means ± the standard deviation (SD) from three independent experiments. Differences between two groups were analyzed by the unpaired/paired Student’s t test (two-tailed tests), and one-way ANOVA followed by Dunnett’s multiple comparisons tests was performed if more than two groups were compared. Data of clinical analysis were indicated as median with the interquartile range. The Mann-Whitney U test was applied for independent samples when the population could not be assumed to be normally distributed. Pearson’s chi-square test was used to analyze the clinical variables. Spearman’s correlation analysis was performed to determine the correlation between two variables. Cumulative survival time was calculated using the Kaplan-Meier method and analyzed by the log-rank test. A multivariate Cox proportional hazards model was used to estimate the adjusted hazard ratios and 95% confidence intervals, and to identify independent prognostic factors. All statistical analysis in this study were performed using SPSS 22.0 software. A P value<0.05 was considered significant.
