# Supplementary material for: Exosomal long noncoding RNA HOXD-AS1 promotes prostate cancer metastasis via miR-361-5p/FOXM1 axis
Source: Cell Death Dis. 2021 Dec 4;12(12):1129. doi: 10.1038/s41419-021-04421-0 (PMC8643358; doi:10.1038/s41419-021-04421-0)
Supplement: Supplementary file 16 — Supplementary Figure Legends [file 41419_2021_4421_MOESM16_ESM.docx]

**Supplementary Figure Legends**

**Figure S1.** (A-B) LNCaP and PC-3 cells were cultured with medium supplied with serum exosomes enriched from localized and metastatic PCa patients (each n=5), and transwell migration assay was used to measure the migration. The picture of patient #1 was displayed as Figure 1A. Scale bar: 200μm.

**Figure S2.** (A-C) LNCaP and PC-3 cells were cultured with medium supplied with serum exosomes enriched from localized and metastatic PCa patients (each n=5), and wound healing assay was used to measure the migration. The results were measured by the percentage of healing compared with baseline and presented as median ± interquartile of values obtained in experiments. Scale bar: 100μm. *p < 0.05, **p < 0.01.

**Figure S3.** (A-B) LNCaP and PC-3 cells were cultured with CRPC cells conditioned-medium and transwell migration assay was used to measure the migration. The results were displayed as relative ratio to control, presented as the means ± SD of values obtained in three independent experiments. Scale bar: 200μm. *p < 0.05, **p < 0.01.

**Figure S4.** Exosomal HOXD-AS1 promotes PCa cell migration *in vitro*. (A) HOXD-AS1 was overexpressed by pCDNA3.1 vector in LNCaP-AI and LNCaP-Bic cells, then cellular and correspondent exosomal HOXD-AS1 was detected by qPCR. The results of real time qPCR were normalized to GAPDH and presented as the means ± SD of values obtained in three independent experiments. (B-C) Stable HOXD-AS1 knockdown LNCaP-AI and LNCaP-Bic cells were constructed by lentiviral transduction. Then the LNCaP and PC-3 cells were incubated with either the HOXD-AS1 knockdown or control exosomes, HOXD-AS1 expression was detected by qPCR. The results of real time qPCR were normalized to GAPDH and presented as the means ± SD of values obtained in three independent experiments. (D-E) HOXD-AS1 knockdown LNCaP and PC-3 cells were treated with either PBS or CRPC exosomes for 48h, the cellular expression of HOXD-AS1 was detected by real time qPCR. The results were normalized to GAPDH and presented as the means ± SD of values obtained in three independent experiments. (F) CD81 and TSG101 were detected by Western Blot in CRPC exosomes and HOXD-AS1 depleted CRPC exosomes. (G) LNCaP and PC-3 cells were treated with either purified HOXD-AS1 knockdown exosomes or control exosomes for 48h, then cellular motility was evaluated by wound healing assay, PBS were used as negative control. Scale bar: 200μm. *p < 0.05, **p < 0.01.

**Figure S5.** Representative images of immunohistochemistry (IHC) staining using firefly luciferase (Luc) antibody of the bone sections. Brown signal indicates positive staining of Luc. Black dot-circled areas indicate the metastatic tumor in the bone. Black scale bars: 500μm, red scale bars: 200μm.

**Figure S6.** HOXD-AS1 regulates miR-361-5p expression in PCa cells. (A-C) The correlation of miR-361-5p with other lncRNAs from published article^1, 2, 3^ were evaluated using TCGA cohort. (D-E) The correlation of HOXD-AS1 with other miRNAs from published article^4, 5^ were evaluated using TCGA cohort. (F-G) HOXD-AS1 knockdown LNCaP and PC-3 cells were treated with either PBS or CRPC exosomes for 48h, the cellular expression of miR-361-5p was detected by real time qPCR. The results were normalized to U6 and presented as the means ± SD of values obtained in three independent experiments. (H) HOXD-AS1 was overexpressed in LNCaP and PC-3 cells, and the HOXD-AS1 and miR-361-5p expression was detected by qPCR. The results of real time qPCR were presented as the means ± SD of values obtained in three independent experiments. HOXD-AS1 expression was normalized to GAPDH and miR-361-5p expression was normalized to U6. (I) HOXD-AS1 was knockdown by lentivirus in LNCaP and PC-3 cells, and the HOXD-AS1 and miR-361-5p expression was detected by qPCR. The results of real time qPCR were presented as the means ± SD of values obtained in three independent experiments. HOXD-AS1 expression was normalized to GAPDH and miR-361-5p expression was normalized to U6. *p < 0.05, **p < 0.01.

**Figure S7.** Exosomal derived HOXD-AS1 promotes cell migration dependent on FOXM1. (A) FOXM1 expression was knockdown by lentiviral transduction in LNCaP and PC-3 cells, and its expression was detected by real time qPCR. The results were normalized to GAPDH and presented as the means ± SD of values obtained in three independent experiments. (B) The protein expression in FOXM1 knockdown cells was detected by Western Blot, GAPDH was used as internal control. (C-D) FOXM1 depleted LNCaP and PC-3 cells were treated with CRPC derived exosomes, and the transwell migration assay was applied to evaluate the migration of cells. The results were displayed as relative ratio to control, presented as the means ± SD of values obtained in three independent experiments. PBS was used as negative control. Scale bar: 200μm. (E-F) FOXM1 depleted LNCaP and PC-3 cells were treated with CRPC derived exosomes, and the cellular migration was measured by wound healing assay. The results were displayed as percentage of healing, presented as the means ± SD of values obtained in three independent experiments. PBS was used as negative control. Scale bar: 100μm. *p < 0.05, **p < 0.01.

**Figure S8.** The relative expression of serum exosomal HOXD-AS1 between T2 and T3-4 PCa patients (n=71 and 59). The results are presented as medians ± interquartile.

**Figure S9.** ROC curve analysis for evaluating the diagnostic potential of serum PSA for distant metastasis.
