# Supplementary material for: Exosomal long noncoding RNA HOXD-AS1 promotes prostate cancer metastasis via miR-361-5p/FOXM1 axis
Source: Cell Death Dis. 2021 Dec 4;12(12):1129. doi: 10.1038/s41419-021-04421-0 (PMC8643358; doi:10.1038/s41419-021-04421-0)
Supplement: Supplementary file 17 — Supplementary References [file 41419_2021_4421_MOESM17_ESM.docx]

**Supplementary References**

1. Wu YH, Yu B, Chen WX, Ai X, Zhang W, Dong W*, et al.* Downregulation of lncRNA SBF2-AS1 inhibits hepatocellular carcinoma proliferation and migration by regulating the miR-361-5p/TGF-beta1 signaling pathway. *Aging (Albany NY)* 2021, **13**(15)**:** 19260-19271.

2. Gao F, Feng J, Yao H, Li Y, Xi J, Yang J. LncRNA SBF2-AS1 promotes the progression of cervical cancer by regulating miR-361-5p/FOXM1 axis. *Artif Cells Nanomed Biotechnol* 2019, **47**(1)**:** 776-782.

3. Lu WX. Long non-coding RNA MEG3 represses cholangiocarcinoma by regulating miR-361-5p/TRAF3 axis. *Eur Rev Med Pharmacol Sci* 2019, **23**(17)**:** 7356-7368.

4. Dong S, Wang R, Wang H, Ding Q, Zhou X, Wang J*, et al.* HOXD-AS1 promotes the epithelial to mesenchymal transition of ovarian cancer cells by regulating miR-186-5p and PIK3R3. *J Exp Clin Cancer Res* 2019, **38**(1)**:** 110.

5. Wang H, Huo X, Yang XR, He J, Cheng L, Wang N*, et al.* STAT3-mediated upregulation of lncRNA HOXD-AS1 as a ceRNA facilitates liver cancer metastasis by regulating SOX4. *Mol Cancer* 2017, **16**(1)**:** 136.

6. Gu P, Chen X, Xie R, Xie W, Huang L, Dong W*, et al.* A novel AR translational regulator lncRNA LBCS inhibits castration resistance of prostate cancer. *Mol Cancer* 2019, **18**(1)**:** 109.

7. Chen C, Luo Y, He W, Zhao Y, Kong Y, Liu H*, et al.* Exosomal long noncoding RNA LNMAT2 promotes lymphatic metastasis in bladder cancer. *J Clin Invest* 2020, **130**(1)**:** 404-421.

8. Jiang J, Chen X, Liu H, Shao J, Xie R, Gu P*, et al.* Polypyrimidine Tract-Binding Protein 1 promotes proliferation, migration and invasion in clear-cell renal cell carcinoma by regulating alternative splicing of PKM. *Am J Cancer Res* 2017, **7**(2)**:** 245-259.

9. Xie R, Chen X, Chen Z, Huang M, Dong W, Gu P*, et al.* Polypyrimidine tract binding protein 1 promotes lymphatic metastasis and proliferation of bladder cancer via alternative splicing of MEIS2 and PKM. *Cancer Lett* 2019, **449:** 31-44.

10. Chen X, Gu P, Xie R, Han J, Liu H, Wang B*, et al.* Heterogeneous nuclear ribonucleoprotein K is associated with poor prognosis and regulates proliferation and apoptosis in bladder cancer. *J Cell Mol Med* 2017, **21**(7)**:** 1266-1279.

11. Chen Z, Chen X, Xie R, Huang M, Dong W, Han J*, et al.* DANCR Promotes Metastasis and Proliferation in Bladder Cancer Cells by Enhancing IL-11-STAT3 Signaling and CCND1 Expression. *Mol Ther* 2019, **27**(2)**:** 326-341.

12. Chen X, Xie R, Gu P, Huang M, Han J, Dong W*, et al.* Long Noncoding RNA LBCS Inhibits Self-Renewal and Chemoresistance of Bladder Cancer Stem Cells through Epigenetic Silencing of SOX2. *Clin Cancer Res* 2019, **25**(4)**:** 1389-1403.
