# Supplementary material for: Exosomal long noncoding RNA HOXD-AS1 promotes prostate cancer metastasis via miR-361-5p/FOXM1 axis
Source: Cell Death Dis. 2021 Dec 4;12(12):1129. doi: 10.1038/s41419-021-04421-0 (PMC8643358; doi:10.1038/s41419-021-04421-0)
Supplement: Supplementary file 19 — Author Contribution statement [file 41419_2021_4421_MOESM19_ESM.docx]

**Author Contributions**

P.G. and X.L. conceptualized the study, acquired funding support, and revised the final manuscript, Y.J. and H.Z. performed the *in vitro* and *in vivo* experimental, construction of vectors, analyzed and visualized data, and wrote the initial manuscript. Y.C., C.G. and J.C. performed molecular experiment including qPCR and western blot. K.L. and T.L. isolated and characterized the exosomes. C.G., L.Q., B.Z. and P.G. performed luciferase assays, RIP, and RNA ISH, J.S. and Y.J. collected clinical samples and profiles, and finished the follow-up. All authors read and approved the final manuscript.
